# Volume Transition and Phase Coexistence in Polyelectrolyte Gels Interacting with Amphiphiles and Proteins

**DOI:** 10.3390/gels6030024

**Published:** 2020-08-13

**Authors:** Per Hansson

**Affiliations:** Department of Pharmacy, Uppsala University, Box 532, SE-75123 Uppsala, Sweden; per.hansson@farmaci.uu.se

**Keywords:** polyelectrolyte, gel, microgel, network, surfactant, protein, drug, macroion, volume phase transition, phase coexistence, phase separation, hysteresis, swelling, collapse, theory, modeling

## Abstract

Polyelectrolyte gels have the capacity to absorb large amounts of multivalent species of opposite charge from aqueous solutions of low ionic strength, and release them at elevated ionic strengths. The reversibility offers the possibility to switch between “storage” and “release” modes, useful in applications such as drug delivery. The review focuses on systems where so-called volume phase transitions (VPT) of the gel network take place upon the absorption and release of proteins and self-assembling amphiphiles. We discuss the background in terms of thermodynamic driving forces behind complex formation in oppositely charged mixtures, the role played by cross-links in covalent gels, and general aspects of phase coexistence in networks in relation to Gibbs’ phase rule. We also briefly discuss a gel model frequently used in papers covered by the review. After that, we review papers dealing with collapse and swelling transitions of gels in contact with solution reservoirs of macroions and surfactants. Here we describe recent progress in our understanding of the conditions required for VPT, competing mechanisms, and hysteresis effects. We then review papers addressing equilibrium aspects of core–shell phase coexistence in gels in equilibrium. Here we first discuss early observations of phase separated gels and results showing how the phases affect each other. Then follows a review of recent theoretical and experimental studies providing evidence of thermodynamically stable core–shell phase separated states, and detailed analyses of the conditions under which they exist. Finally, we describe the results from investigations of mechanisms and kinetics of the collapse/swelling transitions induced by the loading/release of proteins, surfactants, and amphiphilic drug molecules.

## 1. Introduction

Charged polymer networks have the ability to swell by absorbing water from the surroundings. The hydrogels thus created are soft materials with both intriguing and useful properties. Early workers recognized that hydrogels could function as chemo–mechanical devices [1,2]. For example, the reduction in length (or volume) of a gel following the release of water could be used to lift a weight. Already in 1952, Hill argued that a polyelectrolyte gel, constrained to a constant width, and with a sufficiently strong attraction between the polymer chains, should have a first-order phase transition in its length–force curve [3]. This type of “razor edge” behavior reminded of muscle contraction [4]. Although the mechanism behind muscle action turned out to be completely different, the idea has served as a prototype for artificial muscles. 

Tanaka discovered that also free-swelling gels could undergo a discontinuous volume change in response to small changes of environmental parameter values [5]. The phenomenon, with obvious similarities to phase transitions in polymer–solvent mixtures, has received the name volume phase transition (VPT) [6]. One of many interesting observations made by Tanaka and others was that, at the onset of VPT in a spherical gel, the new phase was born in the outermost layer, and the transition progressed with the old and the new phases in a core–shell arrangement until the phase conversion was completed [7]. For long cylindrical gels, the new phase first appeared at the ends, and the boundary to the old phase moved inward until the entire gel was converted [8,9]. In some respects, the three-dimensional polymer network provides gels with mechanical properties resembling those of solids. It is interesting therefore that the melting of crystalline solids always begins on their free surfaces, even when the temperature is the same throughout the material [10]. However, although the VPT in gels has features in common with the melting of solids, there are important differences. The melted phase appearing on the surface is thermodynamically stable at a slightly lower temperature than the melting temperature of the solid. Hence, melting requires no superheating, and so there is no energy barrier hindering the transition. Pre-melting is a consequence of the surface tension of the solid being larger than the sum of the surface tension of the liquid and the solid–liquid interfacial tension [11]. In contrast, Tanaka and co-workers found [7] that the temperature-controlled collapse transition in poly(acrylamide-co-acrylate) gels took place at a higher temperature than the so-called Maxwell temperature (*T_M_*), the latter being the temperature at which the free energy minima for respectively the collapsed and swollen states have the same value. Likewise, for the reversed process, the swelling transition took place below *T_M_*. The result showed that there are energy barriers involved in both processes responsible for the transition temperature hysteresis. Hysteresis as such is, of course, not unique to VPT in gels. In principle, it can be observed whenever there is an energetic barrier for nucleation of a new phase. The familiar example is the liquid-vapor transition, requiring superheating of the liquid and undercooling of the vapor for the respective transition to take place. 

Onuki pointed out that nucleation in the bulk of macroscopic gels should be practically impossible due to an insurmountable sheer-deformation energy barrier [12]. In principle, in the absence of other channels, VPT would start at the onset of thermodynamic instability by the mechanism of spinodal decomposition. However, Sekimoto demonstrated that prior to bulk instability the new phase could form at the gel surface, and that the conditions required also allowed the phase transition to proceed to completion [13]. Tomari and Doi confirmed the result by kinetic model calculations, and showed that, for large-amplitude VPTs, spherical gels should transform via a core—shell phase-coexistence mechanism, and no kinetic barrier stopping the transition on the way to completion should exist [14]. However, since there is a phase-coexistence cost of having two phases of different degree of swelling in the same network, the transition does not take place at the Maxwell point. The deformation ratios in the directions parallel to the boundary between the phases must vary continuously across the boundary. For the transition to start, the driving force must overcome the extra free energy cost of deforming the surface phase from the preferred isotropic state it would be in if not attached to the core. The cost exists for the swelling transition as well as for the collapse transition, and both contribute to the magnitude of the transition temperature hysteresis. Later, researchers observed transition hysteresis and core–shell separation also for VPTs induced by the interaction with solutes in the surrounding liquid [15]. 

This review focusses on phase transitions and phase coexistence in systems of polyelectrolyte gels interacting with species of opposite charge to the network, such as surfactant micelles, peptides, or multivalent salt ions, where core–shell morphologies frequently appear [16]. In such systems, depletion of the interacting species from the solution takes place during the collapse transition unless the gel is in contact with a very large solution volume. When the amount available of the interacting species is not sufficient to complete the phase transition, gels end up in core–shell states of volume intermediate between the fully swollen and fully collapsed states [17,18,19,20,21]. Are those states equilibrium or metastable states? The question is relevant but not so easy to answer. Gibbs phase rule normally helps sorting out ambiguities encountered when constructing phase diagrams. However, the formulation of the rule frequently found in textbooks of physical chemistry is not directly applicable to gels containing two or more phases [22]. One part of this review discusses the reason for that, and highlights the similarity between the phase equilibrium in gels and coherent phase equilibrium in solids, as noted by Hirotsu [23]. Other parts deal with the related, but different, problem of the equilibrium distribution of phases coexisting in single gels and phase equilibrium in multi-gel systems. A major difficulty encountered when attempting to decide the equilibrium state of such systems is that in a collapsing gel, the species responsible for the collapse enter the outer parts first, and strong interactions may lower their redistribution rate. Even if the phase-separated state would be the equilibrium state, the core–shell state could still be metastable with respect to other distributions of the phases in the gel. The reverse case, of gels having released part of their cargo, involves the same type of problem. One part of the review deals with the latter case, which is particularly important in applications of gels to controlled and sustained release of drugs [24,25].

Polyelectrolyte hydrogels appear in many different types of products and their properties vary considerably depending on the type of application. Since we are focusing on VPT, we will only consider weakly cross-linked networks of flexible polyelectrolyte chains, with mesh size large enough not to exclude any interacting species from entering or redistributing inside the gels. There are many reports on non-uniform distributions of proteins in chromatography beads [26,27] resembling the core–shell phase separations we will be dealing with here. However, the literature on that topic does not fit directly into the present context because the rigidity of those networks typically prevents them from undergoing VPT. Furthermore, the current theory of chromatography belongs to the field of adsorption to interfaces rather than to phase behavior of polyelectrolytes. 

The topic of this review borders many related research fields, some of which have been active for decades. We will briefly discuss results of particular interest from those areas, but have no intention to be comprehensive. The field of polyelectrolyte gel–surfactant interactions was reviewed in year 2006 [16]. For key references to early work in the field, we refer to that article. We will exclusively deal with phase phenomena in gels of macroscopic size. Thus, nanogels and colloidal microgels will not be discussed. The purpose of the present article is two-fold. (1) To review papers about phase transition mechanisms and driving forces behind phase separation in polyelectrolyte gels induced by amphiphiles and proteins. (2) To highlight specific research problems that our lab has focused on recently and how we have addressed them. 

## 2. General Aspects 

### 2.1. Interactions in Mixtures of Opposite Charge

Complex formation between linear polyions and macroions of opposite charge has been studied extensively, both experimentally and theoretically [28,29,30,31,32,33,34]. The macroions of interest have been linear polyion chains, dendrimers, proteins, peptides, nanoparticles, and amphiphilic self-assemblies. In most experimental situations, the polyion and the macroion have been added to the system together with their respective counterions. As a rule, the polyion and the macroion associate to form complexes that can be soluble, but more often they stick together cooperatively to form a concentrated phase in some part of the system. The electrostatic driving force behind the association in the dilute regime is easy to understand as the polyion and macroion can neutralize each other. Compared with the non-associated state, where both species are surrounded by their respective counterions, the arrangement is favored by counterion entropy. However, results from computer simulations suggest that the systems also gain coulomb energy [35,36,37]. It has been more challenging to understand the motive behind phase separation or, more precisely, what makes the dense complex phase more stable than a solution of dispersed complexes. Here, surfactant micelles have been useful as model macroions since they can dissolve and reassemble, thus lowering the risk that systems be trapped in non-equilibrium aggregated or colloidal states. This approach has been particularly well developed by Piculell and co-workers [34,38]. The results suggest that, in systems where the electrostatics dominate the interaction between the polyion and the macroion (micelle), the stability of the complex phase is largely determined by two factors: (1) The good opportunities for their charged groups to come in close contact. (2) Phase separation can take place without creating a markedly uneven distribution of the small ions between phases [39]. The entropic penalty of confining the polyion and the macroion to the complex phase is comparatively small. This is particularly clear for combinations of species of low configurational freedom, such as complexes between DNA and rodlike cationic micelles [40], known to arrange themselves in a hexagonal phase [41]. It is less obvious in mixtures of small spherical micelles and highly flexible polyion chains where entropic confinements effects are larger. The very low water content of the complex phases observed also in the latter systems, suggests that complexes are stabilized by polyion bridging and/or ion correlation forces [42,43]. For surfactants, the equilibrium between micelles and free molecules also plays an important role. Since polyions do not attract single surfactant molecules stronger than simple counterions (unless the polyion backbone is hydrophobic), it is clear that the hydrophobic effect, which is the driving force behind micelle formation, is also what stabilizes polyion–surfactant complexes [44,45]. Interestingly, when phase separation leads to lowering of the fraction of free surfactant molecules in the system, the hydrophobic effect is another factor favoring phase separation over dispersed polyelectrolyte-micelle complexes [22,46]. This is a less recognized effect that will be further described below. On the other hand, when there exist hydrophobic attractions between the polyion and the macroion, the tendency to phase separate decreases [39,47,48]. This is clear from a comparison between polyacrylate (PA) and poly(styrene sulfonate) (PSS) [49]. PA interacts mainly electrostatically with surfactant micelles, and mixtures of PA and cationic surfactants readily phase separate into one concentrated complex phase and one dilute phase [50]. For PSS, the interaction with micelles is both electrostatic and hydrophobic, and mixtures of PSS and cationic surfactants form one-phase solutions of soluble complexes, as long as the polyelectrolyte equivalent concentration is slightly larger than the surfactant concentration [51]. To maximize the hydrophobic contacts between the polyion backbone and the micelles, the micelles distribute uniformly among the polyion chains, and even adjust the aggregation number to accommodate more polyion segments at the micelle surfaces.

### 2.2. Role of Crosslinks

Crosslinking the polyion chains to form a continuous network does not change much the nature of the interaction with macroions on the microscopic scale but has important implications for the phase behavior: (1) The polyion has no translational entropy. (2) The polyion is excluded from the liquid phase. (3) The crosslinks limit the swelling of the network, and thereby sets a lower limit to the polyion concentration in any phase in the gel. (4) Deformation of one part of the network affects the state of the network in all other parts.

**Figure 1:**
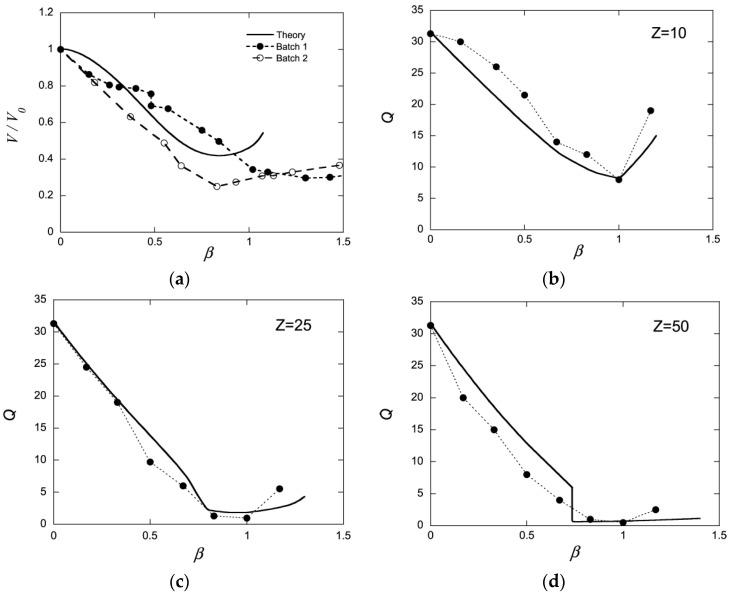
(**a**) Swelling isotherms for a polyacrylate (PA)-microgel in a solution of cytochrome c determined by Jidheden and Hansson (J&H). The microgel volume relative that in protein-free solutions (*V*/*V_0_*) is plotted vs. the protein/network charge ratio (β) in the gel; (**b**–**d**) swelling isotherms for a model system of charged network + spherical macroions of charge as indicated. Symbols are from Monte Carlo simulations by Edgecombe and Linse [59]. Solid curves in both figures are the predictions by the gel model used by J&H [60]. Reprinted with permission. Copyright 2016 American Chemical Society.

The fourth aspect is associated with the elasticity of gels, making them return to their original shape after deformation (“shape memory”). The elasticity derives to a large extent from the loss of configurational entropy of the network chains as they become extended or compressed when the network is deformed from its relaxed reference state. Polyelectrolyte gels can swell extensively due to the osmotic swelling pressure from the counterions. As a result of the elastic response of the network, the volume decreases when the counterions are replaced by macroions via ion exchange. In weakly crosslinked gels the collapse amplitude can be large. An example is shown in Figure 1a, where the volume of sodium polyacrylate microgels decreased about four times after binding the positively charged protein cytochrome c (net charge: +7). The plot shows how the volume decreases with increasing protein/network charge ratio in the gel (β). However, the volume is a continuous function also of the protein concentration in the solution (not shown). Discontinuous volume transitions seem to require short-range attractive forces, such as hydrophobic interactions between the polymer chains or polyion-mediated attractions between the macroions. It is instructive to compare how respectively PA and PSS gels interact with cationic surfactants. PA gels undergo a discontinuous volume phase transition at a critical surfactant concentration in the liquid solution [15]. For the PSS gels, the volume decreases continuously with increasing surfactant concentration in the liquid [52]. In both cases the behavior is in agreement with the phase behavior of the respective non-crosslinked system (see above). Thus, when there is a deficit of surfactant in the system, PA gels contain a collapsed, micelle-rich phase, and a swollen micelle-poor phase in equilibrium. In contrast, PSS gels are homogeneous at all intermediate loading levels. The non-uniform distribution of micelles in PA gels is an indication that the micelles attract one another and that the complexes would be stable even without crosslinks. In the PSS gels, phase separation is counteracted by the hydrophobic interactions (see above), and the uniform distribution of micelles indicates that the attraction is too weak to overcome the effect of those. This leads to net repulsion between the micelles, and the PSS-micelle complexes would be dispersed in the liquid in the absence of crosslinks. The volume reduction of the PSS gels is still significant, which has been proposed to be due to “folding up” of the polyion chains around the micelles rather than an elastic relaxation of the network [53]. 

The fourth point in the above list also implies that the equilibrium state of a gel becomes inhomogeneous when one part of the network is deformed by an external force. This would be the situation if, e.g., the network at one surface of a gel would be chemically attached to a solid substrate. Recently, field theoretical approaches have been used, sucessfully, to model gels deformed in various ways [54,55]. A similar situation appears in gels having a spherical core network of one type attached to a spherical shell network of a different type. Theoretical modeling by Gernandt et al. [56] showed that, when the core is swollen and the shell collapsed, or vice versa, the lateral and radial strains in the shell network are different, and vary with the distance from the gel centre (the core network is always isotropic of symmetry reasons). An important consequence is that the (average) swelling of the swollen part is smaller and the swelling of the collpase part is larger than if they were separated from each other. In general, the state of the network in the core and the shell are interdependent and functions of the relative amounts of network in the core and shell. This is the case also when two or more phases coexist as a result of intermolecular forces in gels containing only one type of network [22]. The dependence on the relative amounts of the phases is absent in phase equilibria involving fluids only, but is not unique to gels. In coherent phase equilibrium in binary alloys, the lattice parameters of the crystalline phases in contact at the grain boundaries are coupled to each other. The composition of the phases are not independent but become functions of the the relative amounts of them [57]. This alters the common texbook formulation of the Gibbs phase rule. According to Gibbs, the number of degrees of freedom f is the number of independent potentials (temperature, pressure, chemical potentials) that can be changed without altering the number of phases. Phase equilibrium requires uniformity of all thermodynamic potentials. In each phase the number of chemical potentials equals the number of components n. By the Gibbs–Duhem equation the number of independent chemical potentials is reduced by one, so the number of independent potentials are *n* − 1 + 2 = *n* + 1. Each phase is represented by an (*n*+1)-dimensional ‘surface’ in the (*n*+2)-dimensional potential space diagram, a two-phase equilibrium by a n-dimensional ‘line’ of intersection between two surfaces, a three-phase equilibrium by a ‘point’ of intersection between three surfaces, etc. In a system with p phases, we have *f* = *n* + 1 − (*p* − 1) = *n* − *p* + 2 if there are no coherency stresses. However, in coherent solids the equilibrium conditions contain the relative amount of the phases as variables. This means that p-1 additional variables must be specified to define the equilibrium state [57]. The total number of degrees of freedom becomes *f* = *n* − *p* + 2 + (*p* − 1) = *n* + 1. In covalent polymer gels the stresses mediated between the phases due to the crosslinks play a similar role as the coherency stresses in solids. This follows from the requirement that network strains parallel to the interface between two phases must vary continously across the interface [58]. For a gel in equilibrium with a liquid we have *f* = *n* − *p* + 2 + (*p* − 2) = *n*, since p includes the liquid phase [22]. Thus, the number of degrees of freedom is not limited by the number of phases. As a check, let us look at the heat induced VPT in rod-shaped NIPA gels in pure water. The (new) collapsed phase nucleates at the ends and gradually grows with increasing temperature. At each temperature a new phase equilibrium is established and the volume ratio of the collapsed and swollen phase increases monotonically with increasing temperature. At fixed pressure we have, *f* = *n* − 1 = 2 − 1 = 1, in agreement with the observation that one potential can change without changing the number of phases. In conflict with that, the common form of the Gibbs phase rule would give *f* = *n* − *p* + 1 = 2 − 3 + 1= 0. In later sections we will give more examples of the unusual features observed in phase separated gels.

### 2.3. Electrostatic Gel Model

Edgecomb and Linse investigated the interaction between polyion networks and macroions of opposite charge with Monte Carlo simulations [59]. The interactions were described on the level of the primitive model of electrolyte solutions. The systems (network+macroions+microions) were net neutral and the medium had dielectric constant equal to that of pure water at 25 °C. The size of the systems were not large enough to result in macroscopic phase separation, but showed how the components were distributed on microscopic scales and how the swelling of the network depended on the charge density of the components. Figure 1b–d show how the volume of the network relative a reference state (Q) changes as a function of the macroion/network charge ratio (β) for three different macroion charge numbers (Z) (symbols). Shown is also the result from a mean-field theory based on Wall’s theory of rubber elasticity combined with a model of polyion-macroion complexes [60] (solid curves). The latter model has been used to calculate phase diagrams of linear polyion–micelle complexes with good results [61]. It has also been used to model several of the gels systems discussed in the present article including the system cytochrome c -PA microgels in Figure 1a (solid curve), and it is therefore important to display its deficiencies and merits. In essence, the model describes electrostatic polyion-correlation attractions between the macroions and the entropic force resulting from the confinement of the small ions, network chains, and macroions in the gel, with a correction of the excluded volume effects among the latter described by the Carnahan-Starling equation of state. Around β=1, the crosslinks have negligible effects on the volume for the Z = 25 and Z = 50 macroions, but for the Z = 10 one the components would swell indefinitely (dissolve) in the absence of cross-links.

## 3. Volume Phase Transition (VPT)

**Figure 2:**
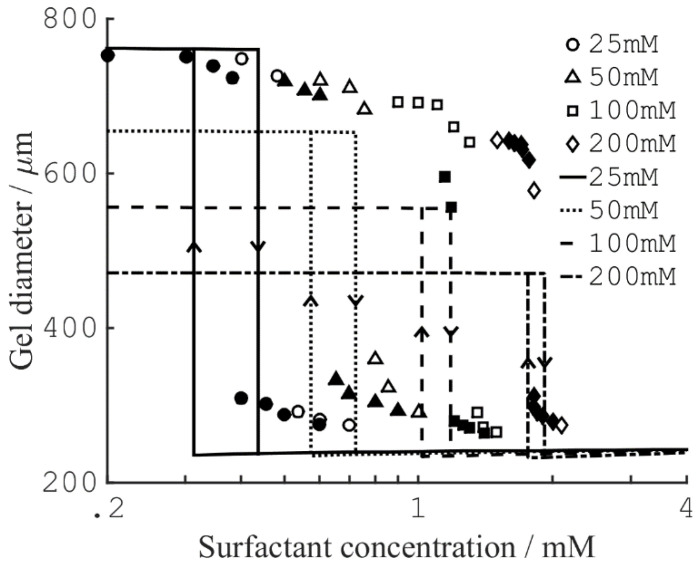
Collapse and swelling transitions in PA gels in solutions of dodecylpyridinium chloride (C_12_PCl) at salt concentrations as indicated. Symbols are experimental data by Sasaki et al. [15] upon increasing (open) and decreasing (filled) the surfactant concentration. Curves are calculated from theory byGernandt and Hansson. Figure is taken from ref. [68]. Reprinted with permission. Copyright 2015 American Chemical Society.

There are many examples in the literature of polyelectrolyte gels collapsing following the addition of multivalent species to the aqueous solution in contact with the gel. In some cases, the gel volume appears to be a discontinuous function of the concentration of the added species. Horkay and co-workers demonstrated that slab gels made of covalently cross-linked PA underwent a discrete volume collapse in aqueous solutions of multivalent inorganic salts [62]. The critical collapse concentration (CCC) was lower for trivalent than for divalent salts but there were also ion specific effects. Hansson et al. observed a similar dependence on charge when short peptides of oligolysine and co-peptides of lysine and alanine interacted with PA microgels [63]. They also found, that decreasing the charge density of the microgel network, changed the behavior from discontinuous to continuous. Several groups have reported of surfactant-induced VPT in polyelectrolyte gels [15,64,65,66,67]. Of special importance is a study by Sasaki et al. [15], who found that the diameter of rod-shaped PA gels varied discontinuously as a function the concentration of the cationic surfactant dodecylpyridinium chloride (C_12_PCl); see Figure 2. The collapse transition in the pre-swollen gels took place at a higher surfactant concentration than the swelling transition in pre-collapsed gels. The hysteresis, as well as the magnitude of the volume change, increased with decreasing concentration of sodium chloride in the solution. These effects will be discussed more in detail later.

**Figure 3:**
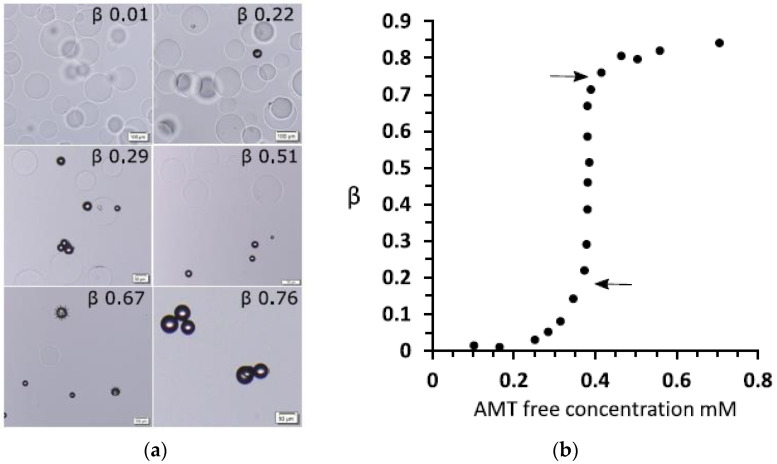
(**a**) Microscopy images of PA-microgels suspended in solutions of the cationic drug amitriptyline hydrochloride and 10 mM NaCl; (**b**) binding isotherm for the same system showing the drug/polyion charge ratio in the microgels (β) plotted vs. the free concentration of drug in the solution. Swollen homogeneous microgels coexisted with homogeneous collapsed microgels in the binding range between the arrows. Data from ref. [69].

When working with large gel specimens, it can be difficult to distinguish between VPT and a very sharp but continuous volume change. One reason is that the liquid volume must be much larger than the gel volume to ascertain that the concentration in the liquid does not change when the molecule responsible for the transition is exchange between the gel and the liquid. Another is that the rate of mass transfer to the gel of the collapsing species can be low near the transition point, which for large gels means very long equilibration times. Here, microscopy studies of spherical microgels with diameters in the order of 100 µm have proven useful. Recently, collapsed and swollen microgels were found to be in equilibrium in a solution of the cationic amphiphilic drug amitriptyline hydrochloride (AMT) [69]. The fraction of collapsed microgels increased with increasing total amount of AMT in the system but at a constant concentration of AMT in the liquid phase; see Figure 3. The absence of semi-swollen gels in the suspension indicated that the volume transition was discontinuous. 

### 3.1. Mechanisms

Since associative phase-separation is a well-known phenomenon in mixtures of polyions and macroions of opposite charge, the mechanism of VPT and its relation to the hysteresis is, perhaps, more interesting than the discontinuity of the transition as such. In principle, nucleation of a new phase in the bulk of a gel would create stresses in all parts of the network. Onuki showed that the activation energy scales with the volume of the gel, and concluded that nucleation involving a large volume change would be practically impossible in a gel of macroscopic size [12]. For macroion-induced transitions in polyelectrolyte gels, the transition mechanisms discussed in the literature are bulk instability and core–shell phase separation. Microphase separation, which is related to the former, has been observed in weakly charged gels with short-range attraction between the polymer chains [70,71]. We will not discuss it here, but it is interesting to note that the volume changes induced by surfactants in PSS gels [16] show some resemblance to the multi-stepwise nature of microphase separation transitions. 

**Figure 4:**
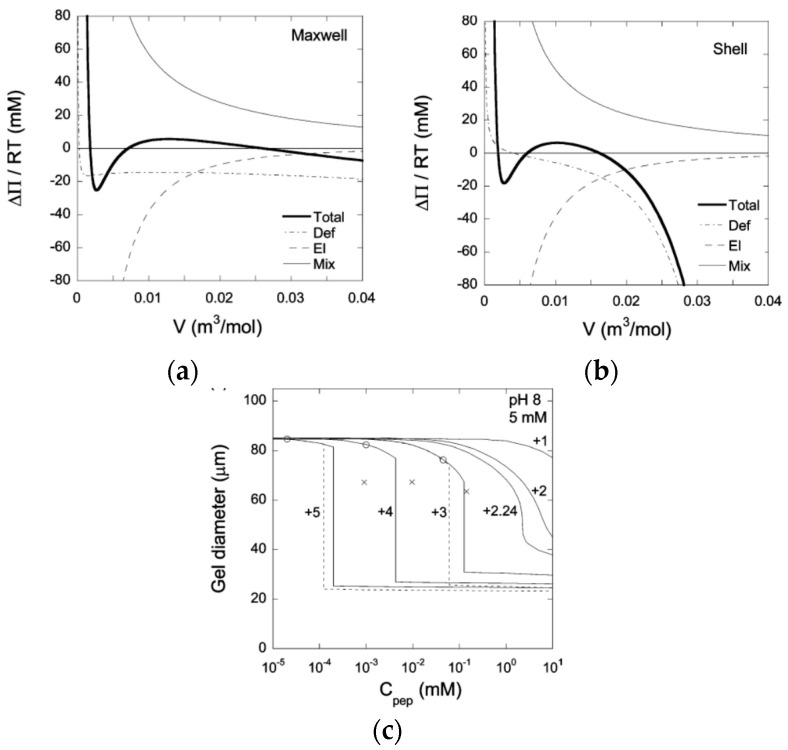
Theoretical investigation of peptide-induced volume phase transitions (VPT) in PA microgel. (**a**) Calculated total and individual contributions to the osmotic pressure difference between gel and liquid (ΔΠ) as functions of gel volume per mole of monomers in the network (V) at the Maxwell point; (**b**) same type of plot as in (**a**) valid at the shell transition point. Peptide charge: +3; 5 mM salt; pH 8. Key to contributions: Tot = total osmotic pressure, Def = elastic deformation energy, El = electrostatic energy, Mix = entropy of mixing (network + peptide + small ion). (**c**) Solid curves: Swelling isotherms calculated for peptides with different charge numbers as indicated. Circles: Maxwell points. Crosses: bulk instability points. Dashed curves: results for peptides of a different length. Figures from ref. [63]. Reprinted with permission. Copyright 2012 American Chemical Society.

In mean field theory, equilibrium between two homogeneous phases is easily determined by means of a Maxwell construction. Figure 4a shows a plot of the osmotic pressure difference between the gel and the liquid as a function of the volume of the gel (per network charge), taken from a theoretical calculation of PA gels in a solution of an oppositely charged peptide [63]. Phase equilibrium requires equal areas under and above the curve in the loop region. The gel volume of the collapsed and swollen state, respectively, are located where the stable branches of the curve intersect the abscissa; the middle intersection point is unstable (dΠ/dV>0). If nucleation in the bulk is prohibited, the collapse transition requires a higher peptide concentration. Following a slow increase of the peptide concentration, the transition would start when the bulk modulus vanishes, i.e., when the osmotic pressure difference at the local maximum of the curve approaches zero. As an alternative, core–shell separation starts when the first thin surface phase becomes stable. The latter concentration can be determined by applying the equal areas rule to a gel free to swell in one direction but forced to have the same strain in the other two directions as the mother phase. Figure 4b shows the result from a calculation on the same system as in Figure 4a. Figure 4c shows swelling curves calculated for peptides of different charge taken from the same study. Circles and crosses mark the (forbidden) Maxwell point and the bulk-instability transition-point, respectively. According to the calculation, core–shell transition is favored over bulk instability. However, the transition points merge with decreasing peptide charge. Interestingly, bulk instability preceded shell formation when the model was applied to the system of di- and trivalent inorganic (micro-) ions studied by Horkay et al. [62].

#### 3.1.1. Spherical Macroions 

**Figure 5:**
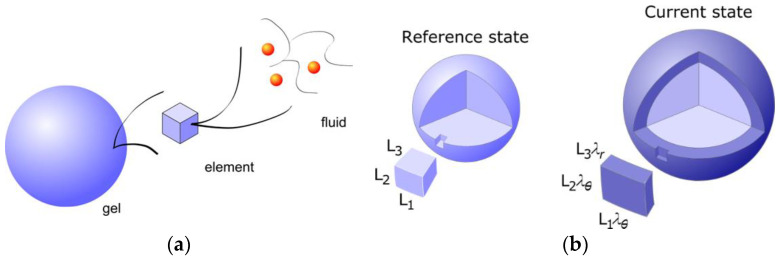
(**a**) A spherical gel described as a collection of small volume elements containing the molecular fluid; (**b**) a gel in the reference state (left) and in the current biphasic state (right). In the latter state, the deformation of the volume elements in the shell in the radial direction (*λ_r_*) is different from that in the circumferential directions (*λ_θ_*). Figure taken from ref. [74]. Reprinted with the permission of AIP Publishing.

Gernandt and Hansson (G&H) used a field theoretical approach to investigate the VPT in spherical polyelectrolyte gels interacting with spherical macroions [72] (Figure 5). They constructed a free energy functional for a system of a gel exchanging molecules with a solution reservoir in the form of an integral over the volume of the gel in a reference state. The functional was shown to depend only on the deformation gradient tensor of the network and the chemical potentials of the components in the reservoir. After minimization with respect to the deformation field, they obtained the same core–shell equilibrium conditions as previously derived for gels in a pure solvent [73]. G&H calculated the free energy density by means of a mean-field theory based on Wall’s theory of rubber elasticity combined with a model of polyion-macroion complexes used earlier [61] to calculate phase diagrams of linear polyion–micelle complexes with good results; see Section 2.3. In the model, the coulomb energy gives rise to cohesive electrostatic forces opposed by the entropic force resulting from the confinement of the macroions, the small ions, and the network chains in the gel. 

**Figure 6:**
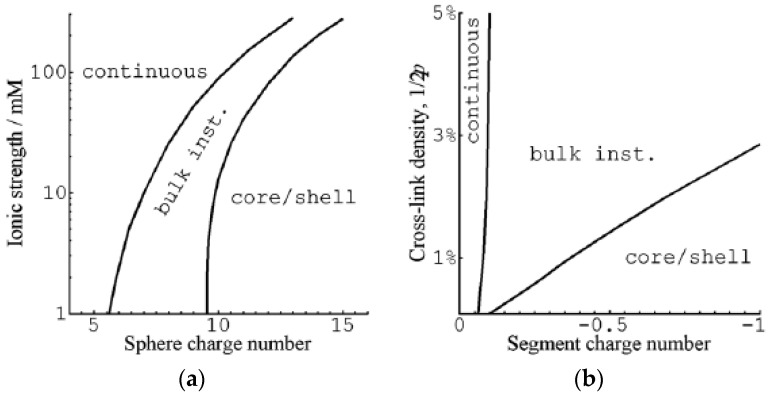
Effect of parameters on the VPT mechanism in systems of spherical gels in large solutions of spherical macroions calculated by G&H. (**a**) Calculated at cross-link density 1% and −1 charge per chain segment; (**b**) at sphere charge +12 and 40 mM salt. [72] Reproduced by permission of the Royal Society of Chemistry.

G&H presented calculations for macroions of size comparable to small globular proteins and surfactant micelles. The charge of the network was varied by changing the charge of segments in the model chains from zero to −1 with the highest charge corresponding approximately to the linear charge density of fully ionized PA. Figure 6 summarizes how the sphere charge number, ionic strength, segment charge number and degree of crosslinking of the network affected the nature of the transition. VPT required a minimum sphere charge, which increased with increasing ionic strength (Figure 6a), and a minimum network charge density (Figure 6b). The dependence on sphere charge is in qualitative agreement with experiments showing, e.g., that at ionic strength 40 mM, the volume transition of weakly cross-linked PA gels is continuous in solutions of cytochrome c (net charge +7), but discontinuous in solutions of cationic surfactants (micelle charge >+50). In spherical PA microgels, it is a well-documented fact that the surfactant-induced VPT occurs by the core–shell mechanism [65,75,76]. The predicted change to the bulk instability mechanism with decreasing electrostatic coupling remains to be tested, but the calculations suggest that the critical charge is closer to that of proteins rather than ionic surfactant micelles. 

**Figure 7:**
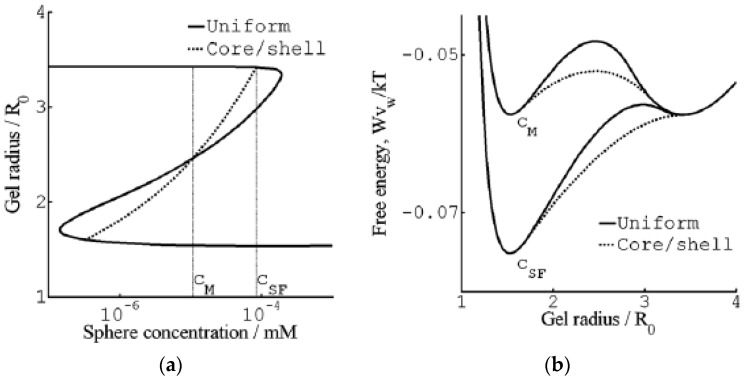
(**a**) Theoretically calculated swelling isotherm for spherical gel in a large solution of macroion with charge +12 and 40 mM salt other parameters as in Figure 6a; (**b**) free energy of the same system as function of gel radius relative the radius in a relaxed reference state (R_0_), for uniform (solid curves) and core–shell states (dotted). [72] Reproduced by permission of the Royal Society of Chemistry.

Figure 7 shows their results from a detailed investigation of the core–shell mechanism for a sphere of charge +12 in 40 mM salt. The solid and dashed lines in Figure 7a are the swelling isotherms for uniform (homogeneous) and core–shell equilibrium states, respectively. The vertical lines mark the sphere concentration in the liquid at the Maxwell point (C_M_) and at the onset of shell formation (C_SF_). Figure 7b shows the free energy (gel + liquid) as a function of gel radius at those concentrations. In both cases, the curves represent states satisfying the equilibrium conditions for respectively uniform and core–shell states in gels with fixed radius. At both concentrations, the core–shell state is more favorable than the uniform state at gel sizes intermediate between the free energy minima for the competing swollen and collapsed states. At the Maxwell concentration, where the two states have equal free energy, an energy barrier hinders the transition. However, the barrier for the core–shell path vanishes exactly at the concentration C_SF_ where the infinitely thin collapsed surface phase forms in equilibrium with the fully swollen network and the solution. Importantly, all intermediate core–shell states are unstable equilibrium states, indicating that there is no thermodynamic barrier preventing the completion of the transition to the uniform collapsed state. This is a generalization of a previous result by Sekimoto [13]. As pointed out by G&H, the dynamics may still give rise to barriers. A theoretical investigation by Tomari and Doi [14] suggested that no such barriers existed that stopped the volume transition of gels in pure solvents once the conditions for the nucleation of the surface phase were fulfilled. This remains to be investigated for the present type of systems, but it can be mentioned that arrested core–shell states have been observed for transitions induced by peptides and aggregating proteins [77,78,79].

We can understand the discontinuous nature of the transition by looking at the changes taking place in a volume element in the shell. Upon nucleation of the shell, the strains in the shell network in directions parallel to the gel surface must remain the same as in the swollen core network. The network is free to adjust in the radial direction but the forces stabilizing the collapsed state limits the swelling. As a result, the shell network becomes non-uniformly deformed, and the volume element has a higher free energy than in a uniform state. As the shell grows, the contractive elastic forces start to affect the core network, which responds by deswelling. This allows, in turn, the shell to relax to a less deformed state with lower (average) free energy density. Thus, since the free energy density is largest in the infinitely thin shell, shell growth proceeds downhill until the entire gel has transformed to the collapsed uniform state. 

According to Figure 6b, increasing the crosslinking of the network can change the transition mechanism from core–shell to bulk instability. This is understandable as the elastic work of deforming the collapsed shell increases with increasing crosslinking density, and at some point, the concentration needed for shell formation will be higher than that for bulk instability. 

**Figure 8:**
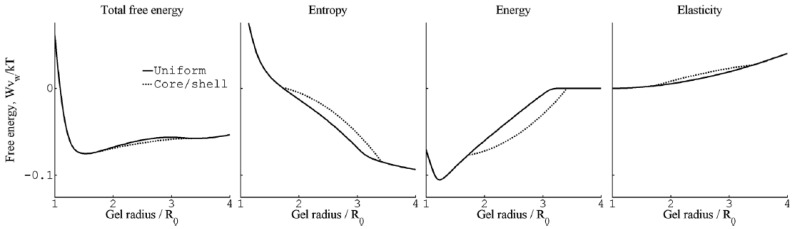
Contributions to the free energy of a quasi-statically compressed gel, comparing the uniform state (solid lines) to the core–shell state (dotted lines). Calculated for the system in Figure 7a, at the concentration where shell formation starts (C_SF_). The free energy components are separated for clarity, but on the same scale. [72] Reproduced by permission of the Royal Society of Chemistry.

Even though the calculations show that the core–shell state is not stable for free-swelling gels in contact with a solution reservoir, G&H found that it had lower free energy than the uniform state at fixed gel sizes intermediate between the fully swollen and the fully collapsed states. An investigation of the thermodynamic driving forces showed that, within the model, the electrostatic energy was solely responsible for the stabilization of the shell. Figure 8 compares the total free energy of the competing states, and the individual contributions from the electrostatic interactions, the entropy of mixing and the elastic free energy due to the entropy of the network chains for spheres of charge +12 in 40 mM salt. As can be seen, only the electrostatics favor core–shell; the entropy and elasticity favor the uniform state. It highlights the importance of distinguishing between the driving force behind binding of macroion to the gel (largely driven by small ion entropy) and the driving force behind phase separation. 

#### 3.1.2. Hysteresis

**Figure 9:**
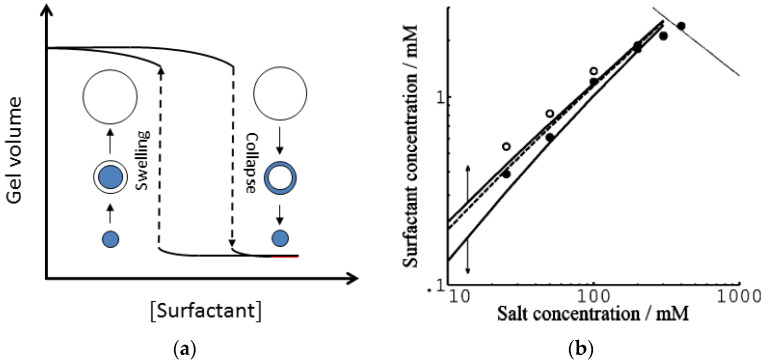
(**a**) Hysteresis in the surfactant-induced volume phase transition. The gel volume shows a jump transition at a critical surfactant concentration in the equilibrium reservoir solution, which is different for the swelling and collapse transitions; (**b**) comparison of experimental and theoretically calculated surfactant concentrations at VPT at different salt concentrations. Symbols: experimental data taken from Figure 2 (open circles: collapse transition; filled circles: swelling transition). Solid curves: theory (arrows up and down indicate the curves for collapse and swelling transition, respectively). Dashed curve: Maxwell transition. Thin dotted line: the calculated critical micelle concentrationin the liquid. Figures taken from ref. [68]. Reprinted with permission. Copyright 2015 American Chemical Society.

For the surfactant-induced VPT in Figure 2, the swelling transition took place at a lower concentration than the collapse transition. Sasaki et al. explained the hysteresis by the bulk instability mechanism [80]. With reference to Figure 7a, that would mean that the transitions occurred where respectively the stable lower and upper branches of the swelling isotherm for the uniform states ends. Indeed, this would be in qualitative agreement with the decrease of the magnitude of the hysteresis with decreasing amplitude of the volume transition. Later, G&H [68] suggested an alternative explanation based on the core–shell mechanism (Figure 9a), supported by time-resolved studies of spherical microgels undergoing volume transition [65,76]. They used the model described above, with charged spheres representing C_12_P^+^ micelles with fixed aggregation number, but allowed for local equilibrium between micellized and free surfactant molecules. To obtain quantitative agreement with experiments, they found that it was necessary to take into account counterion binding to the network charges in the swollen gel state, for which they used the cylindrical Poisson–Boltzmann theory. The result is shown in Figure 9b; the calculated swelling isotherms are included in Figure 2 (solid curves). 

**Figure 10:**
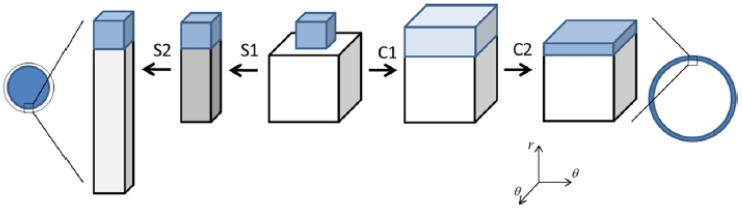
Deformation of shell. The requirement that the network strains in the directions parallel to the core−shell boundary be equal on both sides of the boundary leads to anisotropic deformation of the shell. Middle construct: collapsed (blue) and swollen (gray) volume elements in a hypothetical state without elastic coupling between core and shell. Constructs to the left (swelling transition): biaxial compression (S1) of the swollen shell element to impose the same lateral stretch as in the collapsed core, followed by relaxation (S2) to restore swelling equilibrium. Constructs to the right (collapse transition): biaxial stretch (C1) of the collapsed shell element to impose the same lateral stretch as in the swollen core, followed by relaxation (C2) to restore swelling equilibrium. Figure taken from ref. [68]. Reprinted with permission. Copyright 2015 American Chemical Society.

The swelling transition was found to give the largest deviation from the Maxwell point. Since formation of a very thin surface phase is the critical step for the transition to take place in both directions, the gel boundary and the shell can be treated as locally flat. Thus, it is possible to understand the effect by considering the deformation of flat volume elements on each side of the core–shell boundary, as illustrated in Figure 10. The cubes in the middle represent volume elements of the collapsed and swollen phase in the non-deformed states. As already explained, the new phase forming the shell has to adapt to the core. The path to the right illustrates the adaption of a collapsed shell in two steps. First, the network expands laterally to attain the same strain as in the core. Second, it relaxes in the other direction to attain the volume determined by the intermolecular forces. In the present case, the calculated cohesive forces are so strong that the composition of the shell is nearly the same as in the uniform state. Therefore, the elastic deformation energy largely dominates the free energy penalty of deforming the phase. The path to the left illustrates the corresponding steps for adapting a volume element of a swollen shell to a collapsed core. After the lateral compression in the first step, the volume element relaxes by swelling in the other direction. However, since the expansion is limited to one direction, the swelling forces (dominated by the pressure form the counterions) are too weak to stretch the network enough to recover the volume of the uniform state. Therefore, both the elastic energy and the work of compressing the volume element contribute to the free energy penalty of deforming the volume element from its preferred state. This means that the swelling transition is associated with a larger phase coexistence cost, and therefore a larger deviation from the Maxwell concentration, than the collapse transition, for which the volume change is negligible. 

## 4. Phase Equilibrium in Gels

### 4.1. Shell Composition and Microstructure

In the year 1990, Starodubtzev et al. published the first report of surfactant-rich shells in polyelectrolyte gels [17]. Shortly after, the group of Zezin and Kabanov (Z&K) reported that binding of short polyion chains and proteins could give rise to similar phenomena [18,81,82,83,84,85,86]. The early observations of surfactant-rich shells were made on gels of macroscopic size (~1 mL), that either had been immersed for a short time in a “salt-free” surfactant solution and then transferred to pure water, or stored long times in solutions containing limited amounts of the surfactant. Both types of experiments resulted in dense shells with a sharp boundary to the swollen core. The boundary remained after extended storage times (several months) [87]. These shells were obviously arrested versions of the “new” phase appearing in gels undergoing VPT in reservoir solutions of the surfactant. By arrested we mean that they had stopped growing because the driving force for growing had been removed. After that, they relaxed to a state with composition that may have been different from that in the still growing shell. In our own lab we have occasionally observed that the dense phase initially forming a shell on cylinder-shaped gels, after extended storage times, has relocated to a “waist” flanked by the swollen parts (unpublished data). This is an indication that the core–shell arrangement can be transient depending on the geometry of the gel. However, in many systems studied there is no reason to doubt that there should exist a thermodynamically stable two-phase gel state, even if the core–shell state would not be the true equilibrium state. As evident from previous sections, PA gel interacting with cationic surfactant is one example. In such systems, the interesting question is rather, to what extent the cross-links and the non-uniform network deformations they give rise to affect the composition, microstructure, and stability range of the phases. 

A large number of detailed investigations show that the crosslinks have no or minor influence on the gel microstructure in the homogeneous state after completed surfactant-induced VPTs [21,88,89,90,91,92,93,94,95,96,97,98]. The various types of liquid crystalline phases observed have been reviewed elsewhere [16] and will not be discussed in detail. However, it is clear that the redistribution surfactant is little restricted by the “mesh size” of the network, even in highly cross-linked gels, and that large domains of highly ordered structures can form. Furthermore, the similarity with the microstructure of complexes between surfactants and linear polyelectrolyte [34,38,39,43,99,100,101,102,103], indicates that the cross-links do not affect the conformational freedom of the polyions chains enough to influence the interaction with the surfactant aggregates. The situation may be different in highly deformed shells where the network is under stress. Recall that the lateral strains are largest in the first thin shells formed, and then decreases with increasing fraction of network in the shell. One may thus expect that the composition and microstructure might vary with the amount of surfactant incorporated in the gel. No such effects were described by Z&K, who reported that the composition of the shells were the same as in the fully collapsed homogeneous gels [87]. 

**Figure 11:**
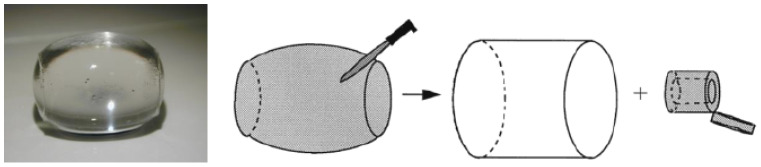
Illustration of the removal of the shell from the core of a PA–C_16_TABr gel (β = 0.5). Photograph to the left shows the intact gel. Figures taken from ref. [21]. Adapted with permission from J. Phys. Chem. B 2002, 106, 9777-9793. Copyright 2002 American Chemical Society.

In a more detailed study, Hansson, Schneider, and Lindman (HS&L) investigated shells formed by C_16_TABr/Cl in PA gels at different levels of surfactant incorporation [21], described by the surfactant/polyion charge ratio in the gel (β). They separated the shell from the core, as illustrated in Figure 11. As a clear demonstration of the elastic effects of phase coexistence, they noted that the shells contracted and the core swelled when placed back into the aqueous solution the gel had been equilibrated in. 

The surfactant/polyion charge ratio in the shells was close to unity for gels with β > 0.2 (shells existed below that range but were not examined). The contraction of shells from gels with β < 0.4 was accompanied by the release of water. For β > 0.4 the water content did not change during contraction, and for β > 0.7 the water content was practically the same as in fully collapsed gels. The variations correlated roughly with changes of the microstructure. Thus, small-angle x-ray scattering experiments showed that the shells had a micellar cubic (Pm3n) phase structure for β < 0.6, and hexagonal for β > 0.6, and at β = 0.6 both phase structures coexisted. However, since only contracted shells were investigated, it is likely that in the intact state before separation from the core, the water-swollen shells at low β had a different microstructure, possibly without liquid crystalline ordering. HS&L concluded that in the lower β-range, the shells behaved qualitatively as solvent swollen networks that absorb liquid upon stretching, and that in the higher β-range, the shells behaved as perfect rubber (no volume change). Results from theoretical calculations by G&H showed [68] that the volume change of shells at low β was larger than expected from changes in network elastic energy. One may speculate that the cohesive forces weakened with increasing deformation of the network, possibly indicating that the polyion-mediated attraction between the micelles depended on the deformational state of the network. However, further investigations are needed to explain the effect and to see if it is general. Here, investigations of the relationship between microstructure and stress–strain properties should be very instructive. Apart from a study by Sasaki et al. [98], where structural changes in surfactant-collapsed gels were found after uniaxial extension, there is little information of that type in the literature.

### 4.2. Composition of Core

**Figure 12:**
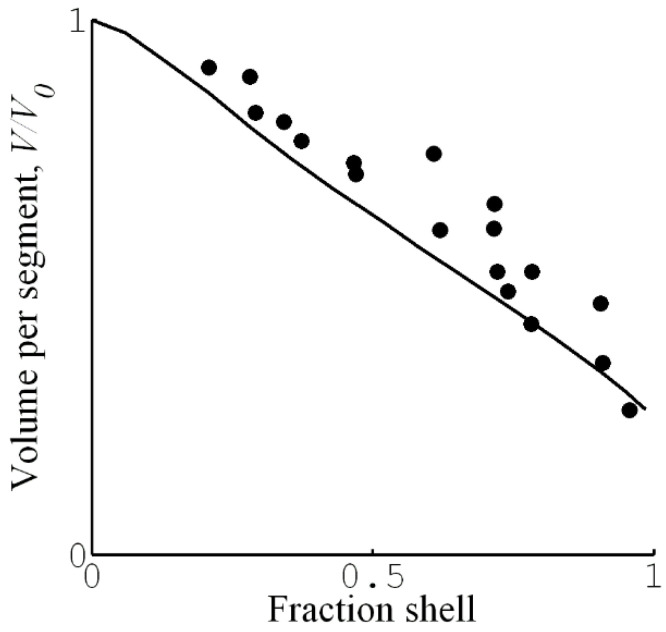
Normalized volume of core per network charge plotted vs. fraction of network in shell for core–shell gels. *V_0_* is the volume per charge in the surfactant-free gel. Symbols: Experimental data for the system PA gel–C_16_TACl [21]. Line: Theoretical model calculation by G&H [74]. Figure taken from ref. [74]. Reprinted with the permission of AIP Publishing.

HS&L investigated how the presence of the surfactant-collapsed shell affected the swelling of the core in PA gels [21]. Figure 12 shows the result in the form of a plot of the swelling ratio of the core plotted vs. fraction of network in shell. The water content of the core decreased with increasing shell fraction. This indicates that the swelling of the core is a function of the proportion of the phases in the gel. As emphasized when discussing the phase rule for gels in Section 2.2, we expect such phase behavior in cross-linked gels but not in systems of linear polyelectrolytes. As a further clarification, we make three remarks. (1) After separation from the shell, the core spontaneously reassumed the swelling ratio of the surfactant-free gel (see Section 4.1). (2) The swelling of the core network is essentially uniform, and so the variations cannot be an effect of non-uniform deformation of the network as such, as appears to be the case for shells. (3) In systems of linear polyelectrolytes, when a complex phase separates out after addition of surfactant, the concentration of polyelectrolyte in the mother phase decreases [104], and may continue to decrease with increasing addition of surfactant until the all polyion chains are in the complex phase. This is opposite to what happened with increasing β in the gels. If one, instead of adding the regular surfactant salt, added the complex surfactant:polyion salt, it would be possible, in principle, to increase the amount of complex phase without depleting the polyelectrolyte solution, but the polyelectrolyte concentration should never increase with increasing fraction of complex phase. 

HS&L interpreted the deswelling of the core as an effect of the elastic free energy of the shell. They also modelled the effect by describing the shell as a stretched rubber membrane imposing a pressure on the core. While capturing the effect on qualitative level, the quantitative agreement was rather poor. Later, G&H showed that the poor agreement was largely an effect of neglecting the variation of the network strains in the directions perpendicular to the gel surface [74]. The solid line in Figure 12 shows the core swelling calculated from the model by G&H (cf. Section 4.3).

Apart from the data in Figure 12, there are few direct measurements of the effect of shells on the swelling of the core in the literature. However, in a recent paper Liang et al. [105] used energy dispersive X-ray spectroscopy to determine the composition of PA-microgels interacting with a cationic antibacterial peptide (net charge: +6). They found that the water content of the core in microgels with peptide-collapsed shells were lower than in peptide-free reference states. The peptide partitioned exclusively to the shell, where the concentration was high but decreased with increasing distance from the core–shell boundary. It is unclear if the system was in equilibrium with respect to the peptide distribution, but the authors concluded that the core was compressed by the deswollen shell, in agreement with the mechanism proposed by HS&L. 

There are very few reports also on the concentration of surfactant in the core of phase-separated gels. HS&L showed by fluorescent probing that the core of the phase separated gels contained negligible concentrations C_16_TA^+^ micelles [21]. Råsmark et al. [106] investigated the composition of PA gels containing a shell formed by C_12_TABr by means of confocal Raman spectroscopy. The surfactant was present at high concentration in the shell but they could not detect any of it in the core. For the very hydrophobic C_16_TA^+^ ions, the low concentration of micelles in the core is expected, since in this case shells exist at very low β. However, for the less hydrophobic C_12_TA^+^ ions, the result is less obvious, since recent results (Section 4.4) suggest that micelles form in the homogeneous gels prior to shell formation. This raises interesting questions about the nature of the phase transition requiring further studies.

### 4.3. Theoretical Modeling of Core–Shell Phase Equilibrium

**Figure 13:**
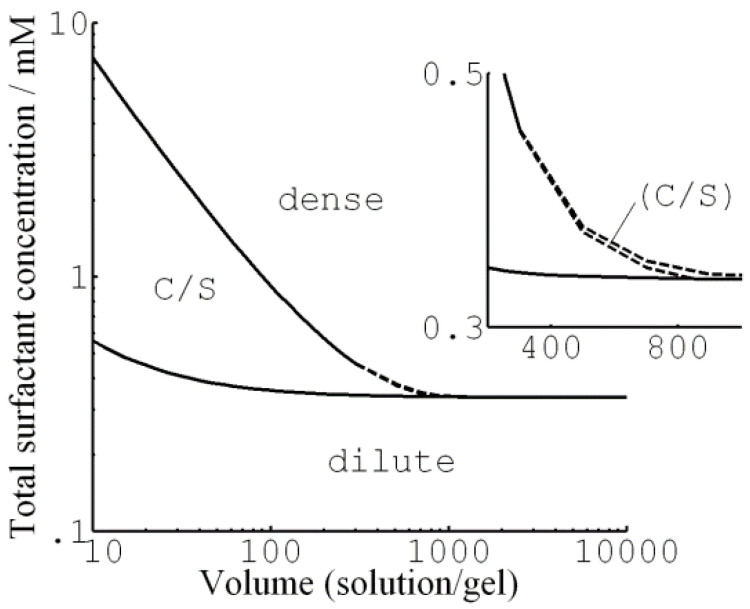
Calculated equilibrium state of the gel upon addition of surfactant to the system. Concentration of simple salt is 10 mM. Volume is the ratio of the volume of the solution to the volume of the gel when it is equilibrated in a large solution of 10 mM of simple salt. C/S denotes phase coexistence between a dense shell and a dilute core. The narrow (C/S) area, enhanced in the inset, is metastable core–shell. Figure taken from ref. [74]. Reprinted with the permission of AIP Publishing.

G&H analyzed theoretically the conditions for surfactant-induced core–shell phase equilibrium in spherical polyelectrolyte gels [74]. To this end, they extended their previous model (Section 3.1.1) to solutions of finite volume containing limited amounts of surfactant. They used the same microscopic model as previously used to model the hysteresis (Section 3.1.2 and Figure 5). Again, they chose parameters relevant for cationic dodecyl-chain surfactants interacting with fully charged PA networks, but adjusted the network elasticity parameter to match the crosslinking density of the experimental systems for which core–shell data were available. Figure 13 summarizes the result in the form of a phase map, showing the stability ranges of the swollen homogeneous state (“dilute”), the collapsed homogeneous state (“dense”), and the biphasic core–shell state (“C/S”). At high solution/gel volume ratio (“Volume”) only the homogeneous gel states are stable. The phase border between them is the surfactant concentration at the collapse transition (Section 3.1.2). Here, the variation of the free energy of the system (gel + solution) as a function of gel radius was qualitatively the same as in Figure 7b. As pointed out by the authors, at concentrations below the critical collapse concentration, it should be possible to make the gel switch from the dilute to the dense state along the core–shell route by “pushing” it over the energy barrier by applying an external force.

**Figure 14:**
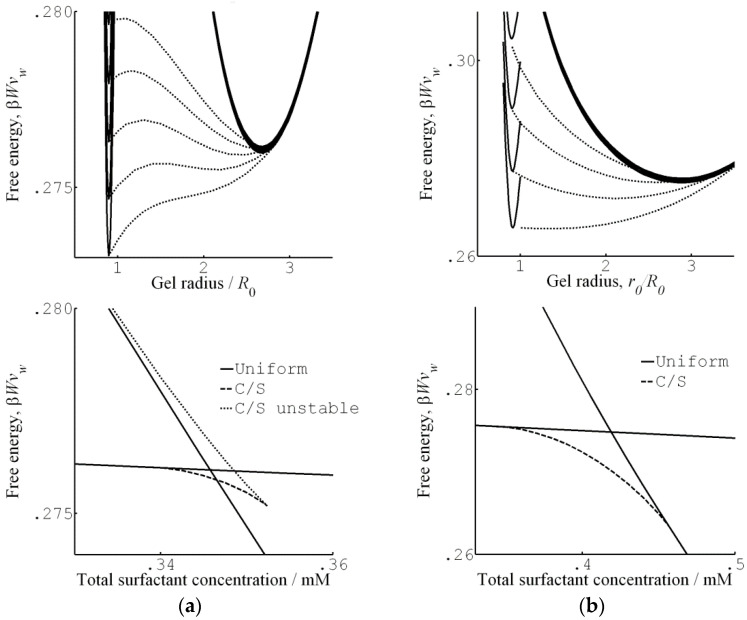
(**a**) Intermediate volume (×700), 10 mM salt. Top: The free energy upon quasi-static volume change, depending on the added concentration of surfactant. Bottom: The locations of the free energy extrema. In a narrow range of concentrations, there is a stable core/shell separation; (**b**) small volume (×200), 10 mM salt. Top: The free energy upon quasi-static volume change of uniform (solid) and C/S state (dotted), depending on the added concentration of surfactant (increasing concentration lowers the curves); *β* = 1/kT. Bottom: The locations of the free energy extrema. Core/shell is stable regardless of shell shickness. Figure taken from ref. [74]. Reprinted with the permission of AIP Publishing.

At intermediate volumes, the core–shell coexistence is the preferred state above the critical concentration required for nucleation of the shell, but is stable only for shells up to a certain thickness. Figure 14 shows examples of the relevant free energy plots. The middle dotted curve in the upper graph of Figure 14a is for a concentration where the shell is thermodynamically stable. Interestingly, at a somewhat higher concentration the free energy minimum for the core–shell state still exists but is no longer the global free energy minimum. Here, the system prefers the fully collapsed state, but a free energy barrier is hindering the growth of the shell, and so the core–shell state is metastable. However, at an even higher concentration, the barrier vanishes and the gel jumps directly to the dense state. 

At sufficiently small solution volumes, we enter the range where shells of any thickness can be thermodynamically stable. Figure 14b shows that, with increasing surfactant concentration, the global free energy minimum shifts continuously from the dilute state, via the core–shell state, to the dense state.

So far we have only described gels with collapsed shells and swollen cores. G&H investigated also the inverted structure, i.e., swollen shells and collapsed core. However, although the difference was small, they found that the free energy of the inverted structure was always higher than for gels with collapsed shells.

**Figure 15:**
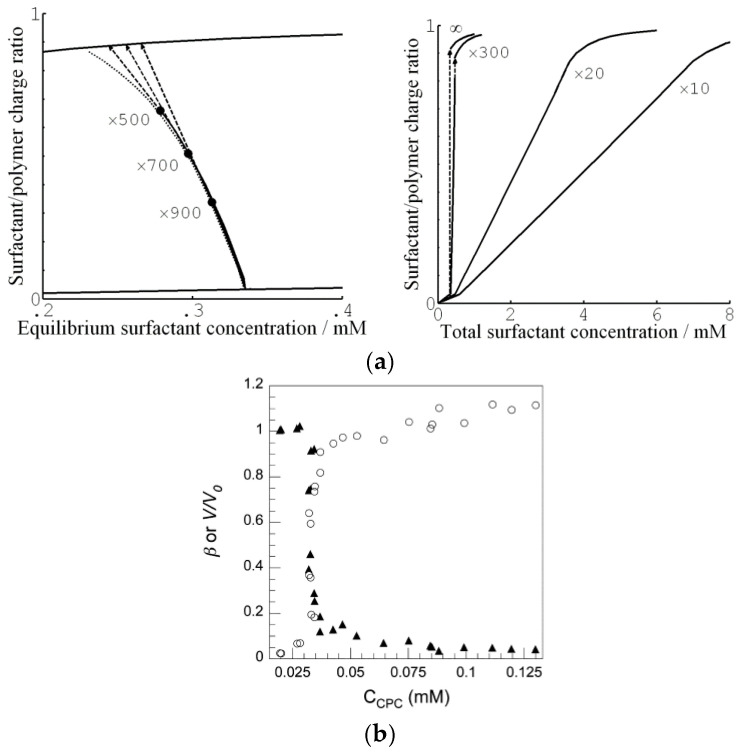
(**a**) Binding isotherms calculated from theory for solution/gel volume ratio as indicated. 10 mM salt. The stable core–shell curves in different volumes are overlapping, also with the unstable curve of infinite volume (dotted). Filled circles indicate points where the shell is destabilized, inducing a jump indicated by dashed arrows. The discrete jump becomes smaller with reduced volume, eventually disappearing entirely. Figure taken from ref. [74]. Reprinted with the permission of AIP Publishing.; (**b**) Experimental binding and swelling isotherms for C_16_PCl–sodium hyaluronate gel in 12 mM TRIS buffer pH 7.4. Acknowledgment: [108].

Figure 15a shows the binding isotherms calculated with 10 mM salt present in the solution. Figure 15a (left) shows the surfactant/polyion charge ratio in the gel (β) as a function of the equilibrium surfactant concentration in the solution at four different solution/gel volume ratios. The concentration of micelles in the gel was found to be negligible prior to shell formation. Therefore, shell formation coincided with the onset of cooperative binding in Figure 15a (right), showing the binding ratio as a function of the total surfactant concentration. For total concentrations higher than that, binding proceeded by the incorporation of surfactant in the shell in the form of micelles. At sufficiently small volume ratios, exemplified by the curves for volume ratios 20 and 10 in Figure 15a (right), the binding isotherm follows the line connecting the branches for the dilute and dense homogeneous states in Figure 15a (left). Notably, the slope of the isotherm in the latter figure is negative, indicating that binding is more favorable the larger the fraction of shell. This is because the free energy density decreases with increasing fraction of shell network since the shell approaches more and more the unperturbed state (i.e., lower phase coexistence cost). This effect directly relates to the VPT and hysteresis in solution reservoirs (Section 3.1.1 and Section 3.1.2). In fact, G&H showed that the same equilibrium relations hold for both situations but the free energy maximum corresponding to the unstable core–shell state in the reservoir becomes a free energy minimum at small volume ratios. This is the reason why the binding isotherms at the larger volume ratios (×500, ×700, ×900) in Figure 15a (left) overlap with the small volume one until the point where the jump transition to the dense homogeneous state takes place (dashed arrows). In fact, that was used as an assumption in an earlier attempt to model core–shell phase coexistence [107]. However, in that study the elastic properties of the shell was not accurately modeled, which resulted in non-physical results at the higher β values. Binding isotherms with negative slope have been reported in the literature, but only rarely [65,108]. One reason could be that the majority of binding isotherms have been determined with little or no extra salt in the system. Under such conditions, the gradual accumulation of salt in the system effectively masks the effect [19]. Figure 15b shows a binding isotherm displaying a negative slope, recorded for C_16_PCl binding to sodium hyaluronate gels [108]. However, the effect is smaller than predicted by theory. As pointed out by G&H, another and perhaps larger flaw was the failure of their model to capture the variations of the water content of the shell. The model accurately predicted the composition of the shells at large β and in the dense homogeneous state, but was way off at low β. The reason for that is unclear. However, G&H showed that the model captured quite well the effect of the shell on the swelling of the core. This is evident from Figure 12, where we have included the result from their calculations (solid curve).

### 4.4. Core–Shell Equilibrium in Microgels

**Figure 16:**
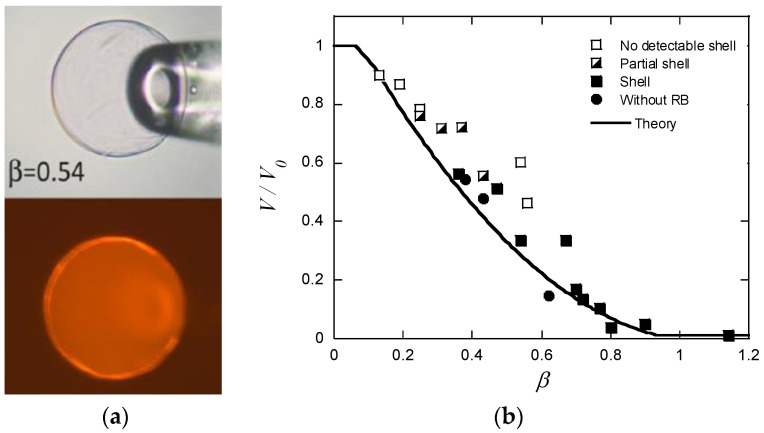
(**a**) Microscopy images of PA-microgel in a solution of C_12_TABr in 10 mM sodium phosphate buffer obtained with the micropipette/micro-droplet technique. Lower figure shows a fluorescence microscopy image with a small amount of a fluorescent dye dissolved in the micelles; (**b**) swelling isotherms for the same system. Microgel volume ratio (V/V_0_) is plotted vs. the charge ratio between the surfactant and network (β). The solid cure is the prediction by the theory of G&H. Data taken from ref. [60]. Reprinted with permission. Copyright 2016 American Chemical Society.

Until recently, reports describing long-lived shells formed by surfactants exclusively dealt with large polyelectrolyte gels. Probable reasons for that are that large gels are easy to prepare and handle, and that it is easy to determine their composition. Furthermore, it is easy to prepare systems matching the conditions for observing stable core–shell states as described in the previous section. The drawbacks with large gels are long equilibration times and the risk of destroying the gels, because of stresses created in the network. A way to avoid such problems is to use microgels in the size range between 10 and 500 µm. It is easy to determine their size and inner morphology with light microscopy, and they typically respond fast to environmental changes. The major difficulty when employing them for studies of core–shell equilibrium is to limit the amount of surfactant available for binding. To handle that, Jidheden and Hansson (J&H) investigated single microgels positioned in small droplets of surfactant solution [60]. By adjusting the size of the droplet and the concentration of surfactant to suite the size of the microgel, they were able to control the total surfactant/polyion ratio in the system. To prevent evaporation, they conducted experiments in hemi-spherical droplets immersed in oil, and inserted the microgels by means of micropipettes. They could then avoid optical effects by monitoring the gels through the flat side of the droplet. Figure 16a shows a PA microgel equilibrated in a solution of C_12_TABr in the presence of 10 mM NaBr. The lower picture is a fluorescence microscopy image with a hydrophobic dye dissolved in the micelles to display the thin shell. The results showed, for the first time, that surfactant-induced shells can be stable in equilibrium in spherical gels. However, they were only able to detect sealed shells covering the entire gel for β-values equal to or larger than ca. 0.4, and gels with β < 0.25 were homogeneous. In the range between those values, the surfactant-rich domains, described as partial shells, covered the gel surface. Figure 16b shows swelling isotherms from the same experiment. The thick solid line is the prediction by G&H’s theoretical model. The rather poor precision of the experimental data precluded quantitative comparison, but the authors concluded that the agreement between theory and experiment was reasonably good in the range were closed shells were detected. Based on the observation of substantial deswelling also in the lower β-range, the authors concluded that a large fraction of the surfactant was present in micelles also in the homogeneous gel state prior to shell formation. This result is conceptually important, since it had not been clear from previous work if micelles could exist at any substantial amount in the swollen gel state. Later, Andersson and Hansson [22] observed the same behavior in specially prepared salt-free systems and gave a theoretical explanation (Section 4.2).

Very recently, Yassir Al-Tikriti has developed an improved set-up by enclosing the system in a glass capillary (unpublished results). This avoids contact with the oil and allows the solution to be taken out for compositional analysis. Furthermore, it markedly improves the precision of the measurements. 

### 4.5. Salt-Free Systems: Cross-Linked Complex Salts

Mixtures of C_12_TABr, NaPA, and water are four-component systems, since mixing generates the component NaBr [104]. The same is true when the polyelectrolyte is covalently cross-linked. Thus, the core–shell gels in equilibrium with a liquid, described in previous sections, are four-component systems with three phases. According to Gibbs’ phase rule the system has one degree of freedom at constant temperature and pressure. This means that it is possible to change the chemical potential of one component, say, C_12_TABr, without changing the number of phases. Thus, even binding isotherms with negative slope for core–shell phase separated gels do not violate Gibbs’ phase rule. In contrast, in a three-component system, it would not be possible, according to the same rule, to change any of the chemical potentials as long as three phases were in equilibrium (at constant temperature and pressure). However, it would not be a violation of the phase rule for gels described in Section 2.2, according to which the number of degrees of freedom is independent of the number of phases. As explained above, this is because of how the equilibrium conditions in the gels depend on the proportion between the phases. 

To be able to investigate three-component systems, Andersson and Hansson (A&H) studied spherical macrogels containing covalently cross-linked complex salts as one component [22], inspired by the three-component phase diagrams developed by Piculell and co-workers for systems containing surfactant ion–linear polyion complex salts [34]. A&H prepared three-component water–C_12_TAPA–NaPA systems by titrating covalently cross-linked PA with mixtures of C_12_TAOH and NaOH to generate systems with different surfactant/polyion charge ratios (β). Here, PA denotes an acrylate segment in a cross-linked polyacrylate network. In principle, C_12_TAPA and NaPA constitute one very large polyelectrolyte, but since they can distribute between phases in the gel, they behave as two components. However, because of the crosslinks they are exclusively in the gel, so the liquid phase is pure water. In addition, they prepared four-component systems by adding the non-ionic surfactant octaethylene glycol monododecylether (C_12_E_8_) to the three-component systems. This was a means to vary the charge of the micelles. A&H found a way to prepare semi-hydrated homogeneous gels containing all components. This allowed them to study how the systems developed after absorbing water, as an alternative to the conventional way by absorbing surfactant from the solution. 

**Figure 17:**
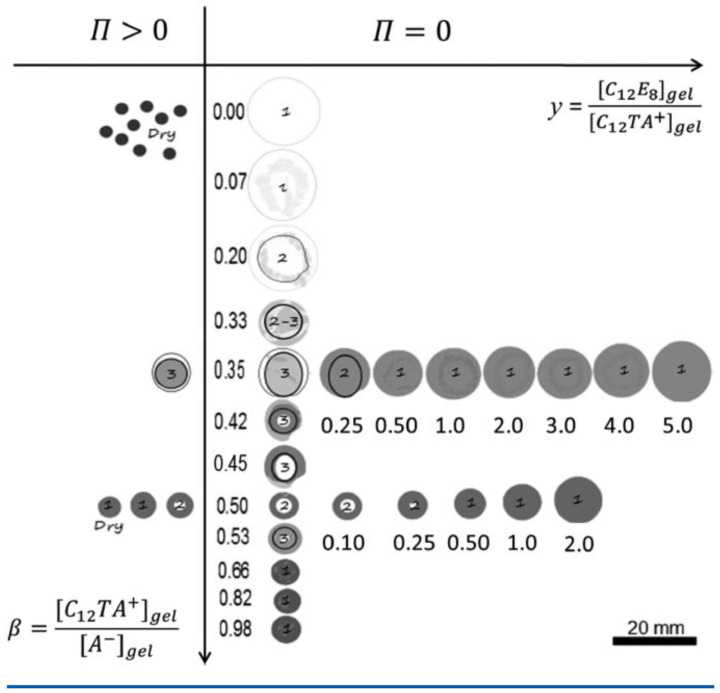
Graphical presentation of the phase behavior of the spherical macrogels investigated by Andersson and Hansson (A&H); see text for details. Figure taken from ref. [22]. Reprinted with permission. Copyright 2017 American Chemical Society.

**Figure 18:**
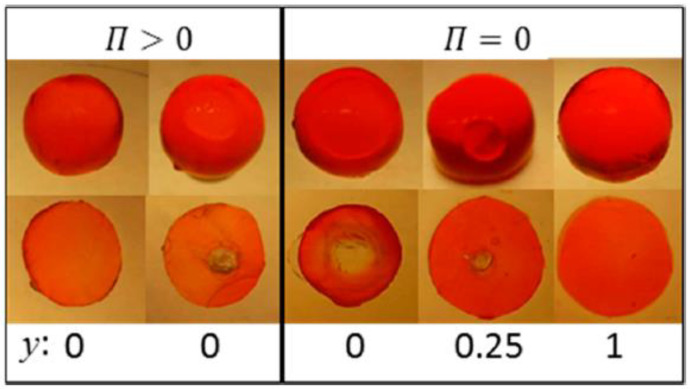
Pictures of intact gels (upper row) and slices through the center of each gel (lower) form the paper by A&H. Pictures to the right of the vertical bar show gels with *β* = 0.5 in equilibrium with pure water (Π = 0) at different non-ionic-to-cationic surfactant ratios (*y*) as indicated by the number under each column. Pictures to the left of the bar show two different gels with *β* = 0.5 and *y* = 0 after drying (Π > 0). The gel to the far left was dried for the longest period; both gels were initially in equilibrium with pure water. Micelle-rich regions are stained red by the presence of small amounts of oil orange in all gels. Figure taken from ref. [22]. Reprinted with permission. Copyright 2017 American Chemical Society.

Figure 17 summarizes the results in the form of a graphical “phase diagram”, where *y* is the molar ratio between non-ionic and ionic surfactant, and β is the surfactant/polyion charge ratio. The cartoons intend to show the inner morphology of gels and their size (drawn to scale). White areas represent micelle-free domains. The decimal numbers refer to the compositional variables β and *y*, and integers are the number of domains in the gel. Data on the right hand side of the vertical axis are for gels equilibrated in pure liquid water (Π = 0); data on the left hand side are for gels equilibrated in vapor with osmotic pressure larger than zero (Π>0). Figure 18 shows photographs of intact gels and slices through the center of gels. Small amounts of a hydrophobic dye was present to probe the distribution of micelles.

#### 4.5.1. Variation of Surfactant/Polyion Charge Ratio

**Figure 19:**
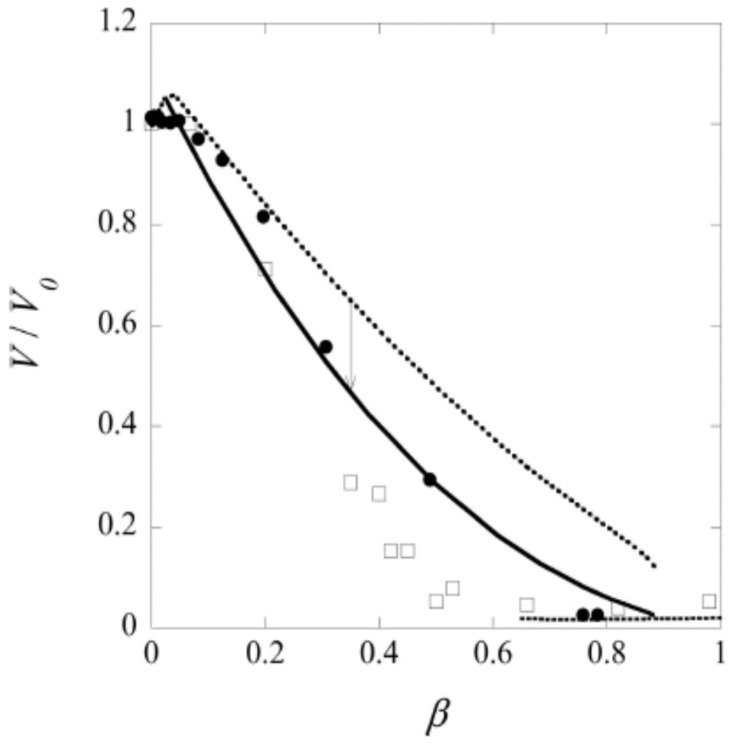
Swelling isotherms. Open symbols are experimental data for NaPA gels with bound C_12_TA^+^ from the study by A&H and filled symbols are experimental data for gels with 1.8% cross-linker prepared in the traditional way published in [19]. Curves are calculated from theory by A&H. The arrow indicates the transition from swollen homogeneous (upper dotted curve) to core–shell (solid curve) states calculated from theory. Reprinted with permission. Copyright 2017 American Chemical Society.

After several months of incubation, the gels in the system water–C_12_TAPA–NaPA (*y* = 0) contained one to three domains. The highly swollen gels with β<0.2 and the collapsed gels with β>0.6 were classified as homogeneous. The semi-swollen gels in the middle range were classified as bi-phasic. As a result of how they were prepared, A&H argued that multi-layered gels with three domains contained a micelle-rich phase “dispersed” in a micelle-lean phase. Phase transition induced by the uptake of water is likely to begin with the formation of a swollen shell. Based on that, the authors suggested that the gels were still undergoing the slow process of phase inversion from dense core/swollen shell to swollen core/dense shell, and thus not in equilibrium. Figure 19 shows the swelling isotherm. The gel volume was nearly constant in the swollen homogeneous state, but decreased rapidly with increasing β in the bi-phasic state, before reaching the level of the dense homogeneous gels. To compare with the surfactant-induced phase transition in pre-swollen gels the authors included data for NaPA gels equilibrated in solutions of C_12_TABr in the same plot. The comparison was considered relevant since the small amount of NaBr accumulating in the solution in the latter system had negligible effect on the swelling. As can be seen in the figure, the behavior was similar for β<0.2, but the conventionally prepared gels were significantly more swollen in the middle β-range, where swollen and collapsed phases coexisted. To interpret the results, A&H included also theoretically calculated swelling isotherms obtained from the gel model described in previous sections. The dotted and solid lines show the predicted swelling of the competing homogeneous and core–shell states, respectively. The arrow indicates where the transition from the swollen homogeneous to the core–shell state would take place according to theory. At higher β, the core–shell state prevails up to β≈ 0.9, where after the collapsed homogeneous is the stable state. Theory captured well the swelling of the homogeneous state at low β. In the middle range, it described quite well the behavior of the collapsed-induced gel bi-phasic states (filled symbols). A&H argued that the swelling-induced bi-phasic states (open symbols) were kinetically arrested non-equilibrium states, which explained their deviant behavior. 

**Figure 20:**
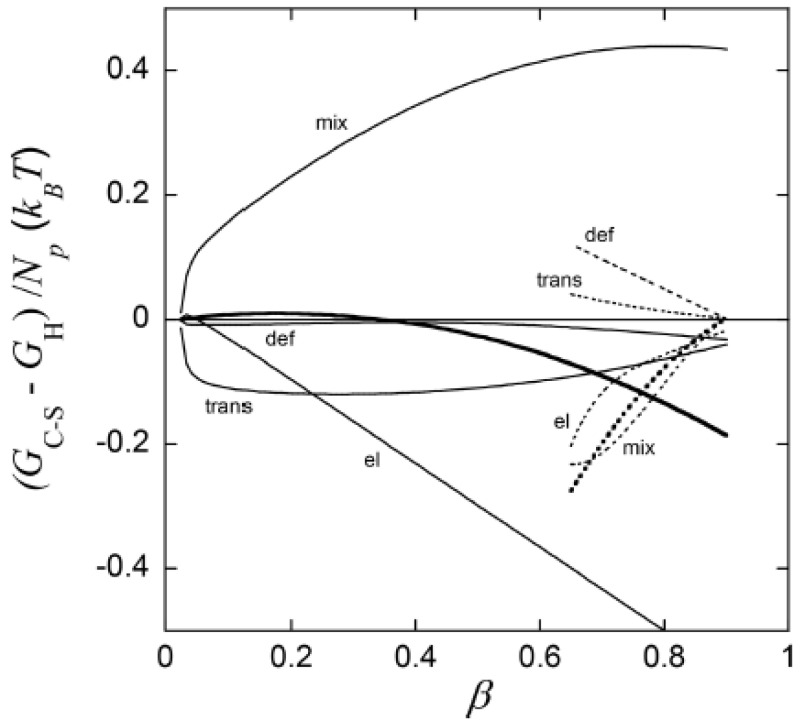
Theoretically calculated total difference in free energy (per polymer charge) between core−shell and homogeneous states (thick curves) plotted vs. the surfactant/polymer charge ratio β (y = 0). Shown are also the contributions to the difference from the free energy of mixing (mix), electrostatic energy (el), elastic deformation energy (def), and free energy of transferring the hydrophobic tails from water to micelles (trans) (thin curves). Figure taken from ref. [22]. Reprinted with permission. Copyright 2017 American Chemical Society.

The most important result from this part of their study was, however, the explanation of the “late” transition to the core–shell state. As noted in Section 4.1, prior to J&H’s microgel study it had not been clear whether micelles could exist at any substantial amount in the swollen gel state, and shell formation was sometimes assumed to coincide with the onset of micelle formation. Figure 20 shows the free energy difference between core–shell and homogeneous states calculated from theory (thick curves), and the individual contributions from the free energy of mixing (mix), elastic deformation energy (def), hydrophobic free energy of transferring the surfactants from water to micelles (trans), and electrostatic free energy (el). A positive difference favors homogeneous and negative favors core–shell. In agreement with the results for spherical macroions (Figure 8), entropy of mixing always favors the swollen homogeneous state. Again, the electrostatic free energy promotes shell-formation (except at very low β), but the hydrophobic effect also gives an important contribution. The reason for the latter is that the fraction of surfactant in micelles is always largest in the core–shell state. However, the hydrophobic effect alone is not strong enough to overcome the entropy of mixing, so transition to the core–shell state can only take place when the electrostatic driving has become sufficiently large. The electrostatic driving force increases in importance with increasing *β* because the electrostatic free energy per micelle is always lower in the core–shell state than in the swollen homogeneous state. A&H noted that the model predicted the onset of shell formation to shift to lower β for surfactants with longer hydrocarbon chains, explaining the difference between C_12_TABr and C_16_TABr. 

According to theory, the collapsed homogeneous state is accessible for β> 0.66. However, it is outcompeted by the core–shell state as long as the latter is accessible, as shown by the dotted curves in Figure 20. Here, entropy of mixing actually disfavors the homogeneous state because the volume of that state is very small. As pointed out by A&H, the β-range where homogeneously collapsed gels were found in the experiments coincide with the range where that state is metastable according to theory. It is possible that an energy barrier could have hindered the swelling of those gels, thus explaining why they did not transform to the core–shell state. Yet, we know little about the mechanism for transitions taking place in that direction. 

#### 4.5.2. Variation of Osmotic Pressure

**Figure 21:**
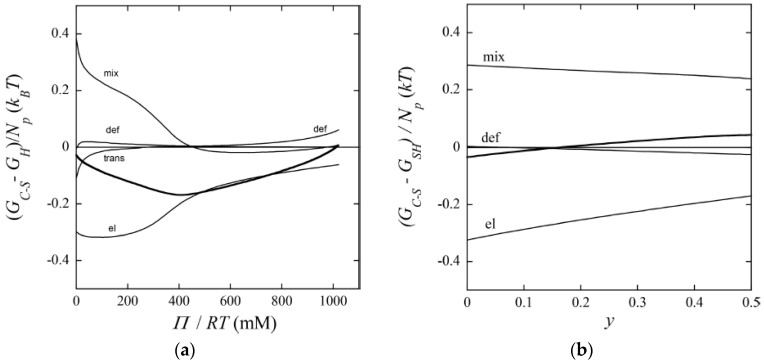
(**a**) Effect of osmotic pressure. Theoretically calculated differences in free energy (per polymer charge) between core−shell and homogeneous states plotted vs. the osmotic pressure for *β* = 0.5; *y* = 0; (**b**) effect of micelle charge. Theoretically calculated total difference in free energy (per polymer charge) between core−shell and homogeneous states plotted vs. *y* for *β* = 0.5. See legend to Figure 20 for more details. Figure taken from ref. [22]. Reprinted with permission. Copyright 2017 American Chemical Society.

The gel with β = 0.50 (*y* = 0) displayed a “classical” core–shell morphology. To study the effect of varying the osmotic pressure, A&H equilibrated the gel in air with relative humidity <100%, which meant osmotic pressures larger than zero. After the first drying steps, the volume of the core had decreased. This showed that it was, indeed possible to change one potential, here the chemical potential of water, without changing the number of phases, something that would violate the regular form Gibbs’ phase rule. However, between two subsequent drying steps, a transition from core–shell to homogeneous state took place, which appeared to be a discrete transition. A&H showed that the transition was reversible by re-incubating the gel at higher relative humidity. Theoretical model calculations could explain the behavior, at least qualitatively. Increasing the osmotic pressure resulted in removal of water almost exclusively from the core. This resulted in transfer of polyelectrolyte network from the core to the shell as a means of the system to maintain a uniform distribution of polyion counterions between the phases and at the same time maintain a low electrostatic energy. However, because of the substantial volume decrease, the difference in entropy of mixing between core–shell and homogeneous gel states vanished. The hydrophobic driving force for phase separation also vanished since the fraction free monomers rapidly decreased for both states. Figure 21a shows that it was the elastic deformation energy, which eventually triggered the transition to the homogeneous state. 

#### 4.5.3. Variation of Micelle Charge

A&H found that decreasing the charge of the micelles could suppress core–shell phase separation, as obtained by increasing the parameter *y*. They studied the effect in samples with β = 0.35 and 0.5 (Figure 17 and Figure 18). The results were clearest in the latter case where the transition took place between *y* = 0.25 and 0.5, which was estimated to correspond to 48 and 40 (+) charges per micelle, respectively. At lower *y*, the fraction of shell increased with increasing *y*, but the gel volume did not change much. Again, theoretical model calculations accounted qualitatively for both observations, and provided the following explanation. Lowering of the micelle charge decreased the electrostatic attraction between micelles, which lead to swelling of the shell structure, and eventually destabilization of the shell. Figure 21b shows the calculated free energy difference between the core–shell and swollen homogeneous states. The calculated micelle charge at the transition point was +53, in reasonable agreement with experiments. However, the model did not capture the rather significant swelling of the swollen homogeneous gels at low charge (cf. Figure 17). A similar discrepancy, although smaller, can be observed when comparing the homogeneous model calculations with Monte Carlo simulations at β = 0.5 in Figure 1b. Clearly, there is room for improvements of the model.

## 5. Volume Transition Kinetics 

### 5.1. Deswelling Kinetics

**Figure 22:**
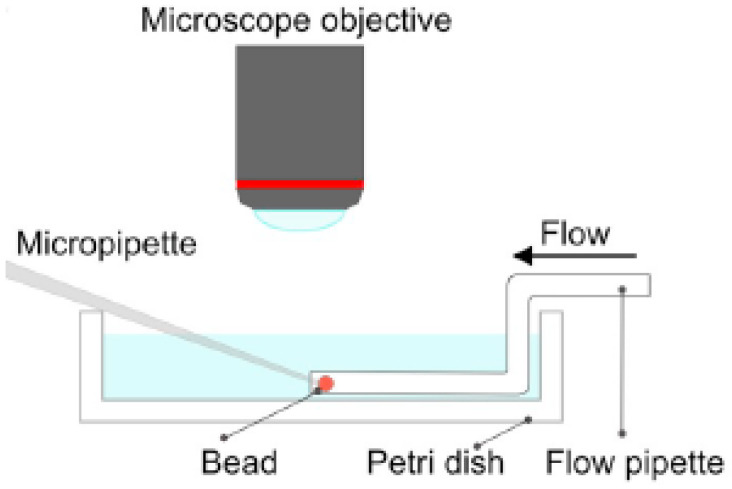
Schematic illustration of the micropipette-assisted microscopy set-up. The bead is positioned inside the flow pipette with a micropipette and the suction of a microinjector enables holding of the bead. The bead is monitored using light or fluorescence microscopy. Acknowledgement: [109].

As already noted, Z&K reported that cationic proteins and surfactants formed dense shells in NaPA macrogels in systems without added salt. In a paper on kinetics [87], they described protein binding as a frontal heterogeneous reaction, and proposed a mechanism by which proteins at the core–shell boundary spontaneously take a step into the core, thereby creating a “vacancy” in the shell. Network counterions fill the vacancy, which migrates outward to the gel boundary where proteins from the solution replace the counterions. They derived a mathematical model of the process in a somewhat sketchy manner, involving first-order rate constants for protein binding/unbinding at the respective boundary. However, what they proposed was essentially an ion exchange mechanism, controlled by inter-diffusion of protein and sodium ions in the shell. Without providing experimental evidence, they claimed that the mechanism applied also to surfactant binding. Furthermore, they claimed that removing the supply of protein or surfactant in the liquid would stop the process and the shell boundary would remain sharp “forever”. We believe maintaining a sharp boundary would require some kind of net-attraction between the molecules in the shell, otherwise redistribution would finally take place, let alone very slowly. Nevertheless, for species forming shells stable at equilibrium at low ionic strength, Z&K’s mechanism of transport through the shell appears to be relevant. In fact, somewhat earlier, Göransson and Hansson [75] had proposed a kinetic model of the surfactant-induced VPT in which the volume change was directly coupled to shell growth. They applied Fick’s first law to describe pseudo-steady state transport of surfactant through the spherical shell, and assumed the core to be “saturated” with surfactant monomers at a concentration equal to the critical aggregation concentration in the gel. They used the stagnant layer diffusion approach to described mass transfer to the gel. To be able to calculate the rate of volume change, they assumed that the transport of surfactant from the bulk liquid to the gel core, via the dense shell, was overall rate determining, and used the equilibrium swelling model for core–shell gels developed by HS&L to describe the gel volume and the shell thickness as a function of β. Nilsson and Hansson (N&H) tested the model by monitoring the volume change of microgels using the micropipette-technique [65]; see Section 4.4. To be able to control the liquid flow rate around single microgels they inserted the gels in a “flow pipette” (Figure 22). 

**Figure 23:**
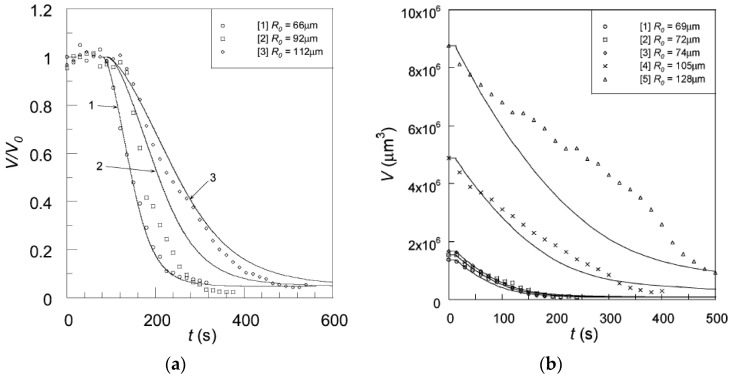
(**a**) Deswelling kinetics for PA-microgels gels of different size in 0.56 mM C_12_TABr, 10 mM NaBr. Relative volume as a function of time for seven different sized gels, with corresponding theoretical lines, is shown. (**b**) Same type of data for PA-microgels in 0.20 mM C_16_TABr, 10 mM NaBr. Figures taken from ref. [65,76]. Figure (**a**) Reprinted with permission. Copyright 2005 American Chemical Society. Figure (**b**) Reprinted with permission. Copyright 2007 American Chemical Society.

The set-up allowed them to maintain a fixed concentration of surfactant in the solution and to calculate the effective stagnant layer thickness surrounding the gel. They investigated how deswelling profiles for NaPA microgels in solutions of C_12_TABr depended on the initial microgel radius, the surfactant concentration, salt concentration, and liquid flow rate. They showed that the model was able to capture quite well how the experimental swelling profile changed with variation of all conditional variables. They used the volume per polyelectrolyte charge in a reference state of the microgel as a single global fitting parameter; all other parameters were set by the experimental conditions or taken from external sources. Figure 23a shows results from a test of the dependence on the gel radius. The authors concluded that stagnant layer diffusion was the rate controlling process during the major part of the deswelling in all cases. In a subsequent study on C_16_TABr loading [76], N&H came to the same conclusion. However, in that case the model failed to describe deswelling profiles under conditions where the relaxation times were long. Figure 23b shows an example, where the model fits are good for small gels but not for the larger ones. The authors were not able to fully explain the effect but suggested that under conditions were particularly dense shells form early in the process, the microstructure imposed a greater resistance to deformation of the shell, and so the assumption behind the model that surfactant transport is rate controlling was no longer valid. To test the model under conditions where the transport through the shell hade a large influence, N&H investigated also macrogels [108]. They found that the model described the form of the C_12_TAB and C_16_TAB deswelling curves, as well as the general time scale, but the quality of the fits were not as good as for microgels. They also described anomalous behaviors, e.g., the formation of “balloon gels” with liquid filled cores.

Recently, Ahnfelt et al. [109,110] investigated the loading of a series of cationic amphiphilic drugs onto commercially available microgel beads (DC Bead), designed for transarterial chemoembolization (TACE), a method used in liver cancer therapy. All systems displayed core–shell phase coexistence during intermediate stages of the loading process, as monitored by light/fluorescence microscopy using the micropipette/flow-pipette technique. The authors showed that stagnant layer diffusion controlled the major part of the deswelling profiles recorded at different drug concentrations [109]. 

Al-Tikriti and Hansson (A-T&H) [69] used the same technique to investigate the deswelling of PA microgels in solutions of amitriptyline hydrochloride, a cationic amphiphilic drug. Following N&H, they varied the microgel radius, drug concentration and liquid flow rate, and observed core–shell coexistence during deswelling under all conditions. They calculated theoretical deswelling curves by means of the steady-state kinetic model described above, with a modified description of transport through the shell. N&H had used the generalized form of Fick’s first law to derive an expression for the transport rate as a function of the difference in chemical potential of the surfactant at the gel boundary and the core/shell boundary, the total surfactant concentration in the shell, and an effective diffusion constant in the shell. For the latter they had used the self-diffusion coefficient for the surfactant in a micellar cubic phase determined by others. To be able to use instead the diffusion constant for the free drug molecule in water (available in the literature), A-T&H described the process as diffusion down the monomer concentration gradient in the shell, governed by a diffusion coefficient set equal to the inter-diffusion coefficient of sodium ions and drug ion, as obtained from the Nernst–Planck equation and the condition of local electroneutrality. They obtained the concentration of free drug molecules in local equilibrium with micelles in the shell from the G&H thermodynamic gel model. The agreement between the theoretical and experimental deswelling curves was good for small microgels; for larger ones the agreement was good at short times but not at long times. The authors concluded that shell transport and stagnant layer diffusion influenced the loading rate to similar extents. 

Malmsten and co-workers have used the above theoretical framework, with various modifications, to analyze deswelling profiles recorded for microgels in solutions of antibacterial peptides [111,112,113,114]. 

### 5.2. Swelling Kinetics

**Figure 24:**
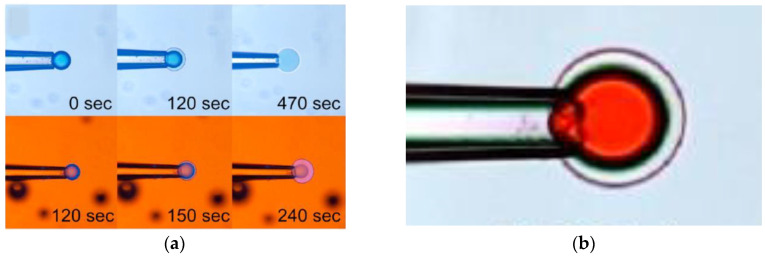
(**a**) Images of DC bead microgels during release of amitriptyline hydrochloride in 150 mM NaCl as observed by light microscopy (upper panel) and fluorescence microscopy (lower panel); (**b**) light microscopy image of DC bead during release of doxorubicin hydrochloride (red color). Acknowledgment: [109].

In their work on amphiphilic drug–microgel interactions, Ahnfelt et al. investigated also the release of the drugs to a medium containing 0.15 M NaCl [109]. Microscopy studies of single microgels in the flow-pipette set-up showed that the microgels swelled upon release. At intermediate stages, a swollen drug-lean shell formed outside the vanishing drug-filled collapsed core. Figure 24a,b shows microscopy images of DC beads releasing amitriptyline hydrochloride and doxorubicin hydrochloride, respectively. The swelling rate correlated with the drug release rate measured in a microgel suspension, and increased systematically with increasing critical micelle concentration of the tested drugs. Based on those observations they proposed that the diffusion of drug monomers through the shell was the rate determining process. 

**Figure 25:**
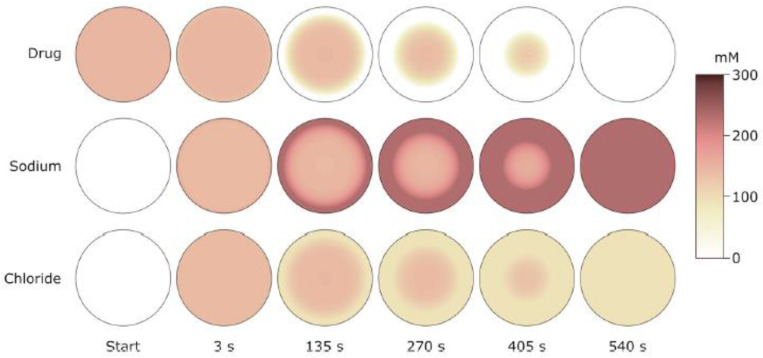
Calculated distributions of amphiphile and salt during release in 150 mM NaCl. Diffusion coefficient of the amphiphile molecule is 5 × 10^−6^ cm^2^ s^−1^ and the critical aggregation concentration inside the bead is 0.5 mM. Bead diameter is 150 μm and the concentration of fixed charges is 150 mM. Acknowledgement: [109].

To understand the role played by salt, they devised a theoretical model where the free drug molecules were allowed to be in local equilibrium with (stationary) micelles. To describe the ion fluxes they used the Nernst–Planck equation in combination with Gauss’s law, which allowed them to take into account the electric coupling of the diffusing ionic species. Similar approaches are common in the literature on ion exchange and chromatography [115]. To be able to solve the rate equations they neglected gel volume changes. Figure 25 shows that the model was able to reproduce qualitatively the time evolution of the inner morphology of the microgels, including the formation of a micelle-depleted shell. Importantly, they found that simple salt (NaCl) quickly penetrated the microgels and reached a uniform distribution before any substantial drug release took place. The calculations showed that the diffusion of drug monomers, down a concentration gradient in the shell controlled the release rate, and that the micelle equilibrium was required for the shell to develop. However, in conflict with a mechanism proposed by others [116], ion exchange did not influence the release rate at physiological salt concentrations. 

**Figure 26:**
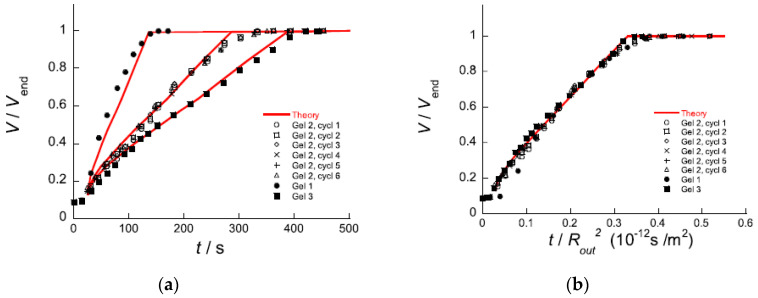
(**a**) Relative microgel volume as a function of time during release of amitriptyline hydrochloride (AMT) for three different microgels of different sizes. Release medium is 150 mM NaCl. V is the actual microgel volume, and V_end_ is the volume after completed release; (**b**) master curve obtained from (**a**) by normalizing time by the square of radius of the respective network in the reference state (R_out_), as suggested by theory. Figure adapted from ref. [69]. Used with permission.

**Figure 27:**
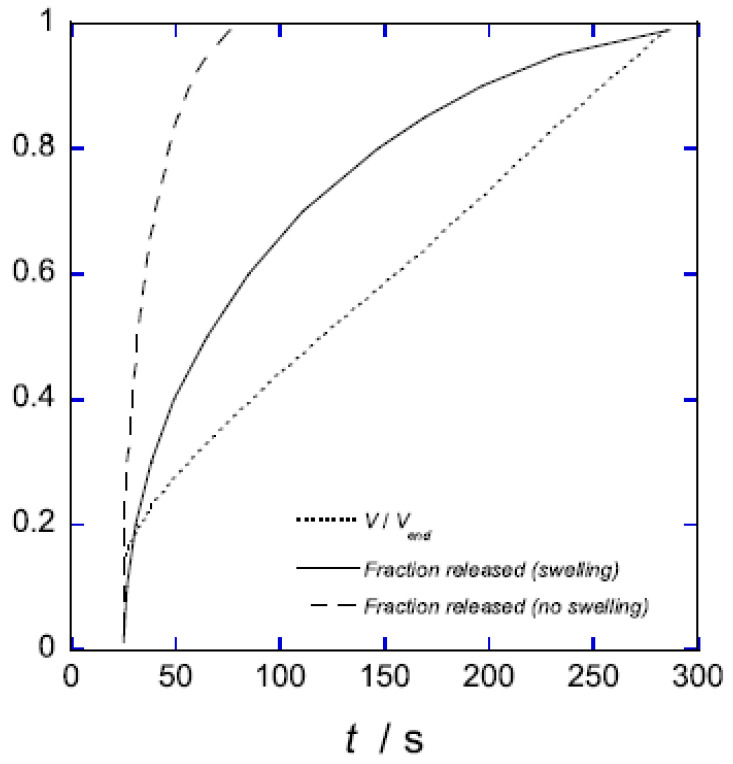
Theoretically calculated fraction of released AMT from a single microgel as a function of time (solid curve). Shown for comparison is the calculated relative gel volume for the same gel (dotted) and the calculated fraction released from a gel with constant radius (dashed). Figure taken from ref. [69]. Used with permission.

The volume of DC beads did not change dramatically during the release of the drugs in 0.15 M NaCl, justifying to some extent that the gel volume was kept constant in the theoretical calculations. To investigate the release mechanism in a system with a larger swelling amplitude, A-T&H studied the release of amitriptyline hydrochloride from highly responsive PA microgels [69]. Single-microgel experiments showed that also in this system a swollen shell coexisted with the dense core at intermediate stages during the release. Since the swelling profiles were largely independent of the liquid flow rate, the authors concluded that the transport processes inside the microgels were rate determining. However, the swelling rate decreased with increasing size of the network, as shown by Figure 26, where Gel 1 is the smallest and Gel 3 is the largest. To model the release kinetics they assumed that the overall rate controlling process was the transport of drug from the core/shell boundary to the solution, via the shell, and applied the pseudo-steady state approximation to derive the rate law from Fick’s first law in spherical geometry. They used the thermodynamic gel model to calculate the core and shell volumes, as well as the concentration of free drug monomers in the shell at the core/shell boundary for quasi-stationary intermediate gel states. As a simplification, they forced the shells to be homogeneous but calculated the strains in the non-uniformly deformed shell network. Figure 26 shows that, with the surfactant diffusion coefficient as the only adjustable parameter common to all curves, the model fitted satisfactory to the experimental data. The good quantitative agreement shows that the modelling of the swelling of the shell and the mass transport through it was reasonably good. The latter aspects are important in applications to drug delivery, where the possibility to modulate the release profiles are important. Figure 27 highlights the effect of shell swelling by comparing with the calculated release for a gel with fixed gel volume. We end by mentioning that ongoing experiments in our lab show that the sharp boundary between the dense core and the swollen shell appears to be stabilized by thermodynamic factors also in the presence of 0.15 M NaCl.

## 6. Conclusions

Our review shows that the research field has progressed slowly but steadily since the previous review appeared in the year 2006. Several fundamental aspects pertaining to the nature of the volume transition induced by proteins and amphiphilic substances in polyelectrolyte gels have been clarified. Increasing charge of the binding species and network chains as well as decreasing ionic strength and crosslinking density, have been found to increase the tendency of gels to undergo discontinuous phase transition and to favor the core–shell mechanism over bulk instability transition. Theoretical model calculations of inhomogeneous gels, in equilibrium or in quasi-static equilibrium states, have provided detailed insight into the phase transition mechanisms and how the systems are expected to develop under various environmental conditions. In particular, net attraction between proteins or micelles in the gel has been found to be a requirement for core–shell phase coexistence, and it has been suggested that the electrostatic interactions, mediated by the polyion chains in the networks, can be solely responsible for that, but for amphiphilic molecules the hydrophobic driving force also contributes. The result highlights the importance of distinguishing between the driving force for phase separation and the driving force for binding to the gels. For (non-hydrophobic) macroions, the entropy of releasing network counterions is a major motive for binding but not for phase separation; for self-assembling molecules the hydrophobic effect is a dominating driving force for binding, but can also be a motive behind phase separation. 

Core–shell phase separation appears to be the dominating mechanism involved in VPTs induced by surfactants and micelle-forming drugs, and experimental as well as theoretical evidence have been provided to show that the phase separated state can be stable in equilibrium. Several interesting effects have been related to the special conditions imposed on the system when phases of different composition coexist in the same elastic network. The hysteresis in the surfactant concentration at the VPT can be related to the phase coexistence cost of nucleating the shell. The magnitude is largely determined by the swelling transition, involving both elastic deformations and volume-related free energy penalties. Core–shell states are stable in equilibrium only when there is a deficit of the collapse-inducing species in the system, but under special conditions the core–shell state can be metastable. Similar to coherent phase equilibrium in alloys, the phase equilibrium conditions are functions of the proportion between the phases. Therefore, the number of degrees of freedom regulating the phase behavior becomes independent of the number of phases in the gel, and the composition of the core and shell changes as the proportions of the phases change.

Finally, important progress has been made regarding our understanding of the mechanisms and kinetics governing the release of micelle-forming amphiphilic molecules from microgels. In particular, an approximate but efficient way to describe diffusive transport through the progressively swelling shell has been found to describe well the swelling profile of microgels releasing cationic drugs. 

A brief look at future directions of the field suggests continued investigations of the mechanism of transitions between homogeneous gel states and core–shell states. This should include transitions between metastable and stable states, as well as the jump transitions between equilibrium states suggested by the theoretical calculations. In relation to the latter, it is important to investigate the concentration of micelles in the swollen parts before and after the transition to the core–shell state, which according to theory changes discontinuously. Other important unanswered questions regard the microstructure of the phases in core–shell separated microgels, which should be investigated for both gels in equilibrium and gels undergoing phase transition.

## References

[1] Katchalsky A. (1949). Rapid swelling and deswelling of reversible gels of polymeric acids by ionization. (A synthetic contractile system). Experientia.

[2] Katchalsky A., Zwick M. (1955). Mechanochemistry and ion exchange. J. Polym. Sci..

[3] Hill T.L. (1952). Some Statistical Mechanical Models of Elastic Polyelectrolytes and Proteins. J. Chem. Phys..

[4] Hill T.L. (1953). Energetics and molecular mechanisms in muscle action. Part II—Statistical thermodynamical treatment of contractile systems. Discuss. Faraday Soc..

[5] Tanaka T. (1978). Collapse of Gels and the Critical Endpoint. Phys. Rev. Lett..

[6] Dušek K. (1993). Responsive Gels: Volume Transitions I.

[7] Matuso E.S., Tanaka T. (1988). Kinetics of discontinous volume-phase transition of gels. J. Chem. Phys..

[8] Hirotsu S. (1988). Critical points of the volume phase transition in N-isopropylacrylamide gels. J. Chem. Phys..

[9] Bai G., Suzuki A. (1999). Phase separation of weakly ionized polymer gels during shrinking phase transition. J. Chem. Phys..

[10] Frenken J.W.M., Marée P.M.J., Van Der Veen J.F. (1986). Observation of surface-initiated melting. Phys. Rev. B.

[11] Dash J.G. (1999). History of the search for continuous melting. Rev. Mod. Phys..

[12] Onuki A. (1988). Paradox in phase transitions with volume change. Phys. Rev. A.

[13] Sekimoto K. (1993). Temperature hysteresis and morphology of volume phase transition of gels. Phys. Rev. Lett..

[14] Tomari T., Doi M. (1995). Hysteresis and Incubation in the Dynamics of Volume Transition of Spherical Gels. Macromolecules.

[15] Sasaki S., Koga S., Imabayashi R., Maeda H. (2001). Salt effects on the volume transition of ionic gel induced by the hydrophobic counterion binding. J. Phys. Chem. B.

[16] Hansson P. (2006). Interaction between polyelectrolyte gels and surfactants of opposite charge. Curr. Opin. Colloid Interface Sci..

[17] Starodubtsev S.G. (1990). Influence of topological structure of polyelectrolyte networks on their interaction with oppositely charged micelle-forming surfactants. Vysokomol. Soedin. Ser. B.

[18] Khandurina Y.V., Rogacheva V.B., Zezin A.B., Kabanov V.A. (1994). Interaction of cross-linked polyelectrolytes with oppositely charged surfactants. Polym. Sci..

[19] Hansson P. (1998). Self-Assembly of Ionic Surfactant in Cross-Linked Polyelectrolyte Gel of Opposite Charge. A Physical Model for Highly Charged Systems. Langmuir.

[20] Hansson P., Schneider S., Lindman B. (2000). Macroscopic phase separation in a polyelectrolyte gel interacting with oppositely charged surfactant: Correlation between anomalous deswelling and microstructure. Prog. Colloid Polym. Sci..

[21] Hansson P., Schneider S., Lindman B. (2002). Phase Separation in Polyelectrolyte Gels Interacting with Surfactants of Opposite Charge. J. Phys. Chem. B.

[22] Andersson M., Hansson P. (2017). Phase Behavior of Salt-Free Polyelectrolyte Gel–Surfactant Systems. J. Phys. Chem. B.

[23] Hirotsu S. (1997). Some exotic properties of polymer gels associated with the volume phase transition. Ferroelectronics.

[24] Lewis A., Dreher M.R. (2012). Locoregional drug delivery using image-guided intra-arterial drug eluting bead therapy. J. Control. Release.

[25] Lewis A., Holden R.R. (2011). DC Bead embolic drug-eluting bead: Clinical application in the locoregional treatment of tumours. Expert Opin. Drug Deliv..

[26] Linden T., Ljunglöf A., Hagel L., Kula M.-R., Thömmes J. (2002). Visualizing patterns of protein uptake to porous media using confocal scanning laser microscopy. Sep. Sci. Technol..

[27] Dziennik S.R., Belcher E.B., Barker G.A., DeBergalis M.J., Fernandez S.E., Lenhoff A.M. (2003). Nondiffusive mechanisms enhance protein uptake rates in ion exchange particles. Proc. Natl. Acad. Sci. USA.

[28] Kizilay E., Kayitmazer A., Dubin P.L. (2011). Complexation and coacervation of polyelectrolytes with oppositely charged colloids. Adv. Colloid Interface Sci..

[29] Thünemann A.F., Müller M., Dautzenberg H., Joanny J., Löwen H. (2004). Polyelectrolyte complexes. Adv. Polym. Sci..

[30] Ulrich S., Seijo M., Stoll S. (2006). The many facets of polyelectrolytes and oppositely charged macroions complex formation. Curr. Opin. Colloid Interface Sci..

[31] Cooper C., Dubin P.L., Kayitmazer A., Turksen S. (2005). Polyelectrolyte–protein complexes. Curr. Opin. Colloid Interface Sci..

[32] De Kruif K.G., Weinbreck F., De Vries R. (2004). Complex coacervation of proteins and anionic polysaccharides. Curr. Opin. Colloid Interface Sci..

[33] De Vries R., Stuart M.C. (2006). Theory and simulations of macroion complexation. Curr. Opin. Colloid Interface Sci..

[34] Piculell L. (2013). Understanding and Exploiting the Phase Behavior of Mixtures of Oppositely Charged Polymers and Surfactants in Water. Langmuir.

[35] Wallin T., Linse P. (1996). Monte Carlo Simulations of Polyelectrolytes at Charged Micelles. 1. Effects of Chain Flexibility. Langmuir.

[36] Wallin T., Linse P. (1996). Monte Carlo Simulations of Polyelectrolytes at Charged Micelles. 2. Effects of Linear Charge Density. J. Phys. Chem..

[37] Wallin T., Linse P. (1997). Monte Carlo Simulations of Polyelectrolytes at Charged Micelles. 3. Effects of Surfactant Tail Length. J. Phys. Chem. B.

[38] Svensson A., Piculell L., Cabane B., Ilekti P. (2001). A New Approach to the Phase Behavior of Oppositely Charged Polymers and Surfactants. J. Phys. Chem. B.

[39] Dos Santos S., Gustavsson C., Gudmundsson C., Linse P., Piculell L. (2011). When Do Water-Insoluble Polyion−Surfactant Ion Complex Salts “Redissolve” by Added Excess Surfactant?. Langmuir.

[40] Leal C., Moniri E., Pegado L., Wennerström H. (2007). Electrostatic Attraction between DNA and a Cationic Surfactant Aggregate. The Screening Effect of Salt. J. Phys. Chem. B.

[41] Gelbart W.M., Bruinsma R.F., Pincus P.A., Parsegian V.A. (2000). DNA-Inspired Electrostatics. Phys. Today.

[42] Granfeldt M.K., Joensson B., Woodward C.E. (1991). A Monte Carlo simulation study of the interaction between charged colloids carrying adsorbed polyelectrolytes. J. Phys. Chem..

[43] Svensson A., Piculell L., Karlsson L., Cabane B., Jönsson B. (2003). Phase Behavior of an Ionic Surfactant with Mixed Monovalent/Polymeric Counterions. J. Phys. Chem. B.

[44] Hayakawa K., Kwak J.C.T. (1982). Surfactant-polyelectrolyte interactions. 1. Binding of dodecyltrimethylammonium ions by sodium dextran sulfate and poly(styrenesulfonate) in aqueous solution in the presence of sodium chloride. J. Phys. Chem..

[45] Hansson P. (2001). Self-Assembly of Ionic Surfactants in Polyelectrolyte Solutions: A Model for Mixtures of Opposite Charge. Langmuir.

[46] Tararyshkin D., Kramarenko E., Khokhlov A. (2007). Two-phase structure of polyelectrolyte gel/surfactant complexes. J. Chem. Phys..

[47] Magny B., Iliopoulos I., Zana R., Audebert R. (1994). Mixed Micelles Formed by Cationic Surfactants and Anionic Hydrophobically Modified Polyelectrolytes. Langmuir.

[48] Guillemet F., Piculell L. (1995). Interactions in aqueous mixtures of hydrophobically modified polyelectrolyte and oppsitely charged surfactant. Mixed micelle formation and associative phase separation. J. Phys. Chem..

[49] Hansson P., Almgren M. (1994). Interaction of alkyltrimethylammonium surfactants with polyacrylate and poly (styrensulfonate) in aqueous solution: Phase behavior and surfactant aggregation numbers. Langmuir.

[50] Thalberg K., Lindman B., Bergfeldt K. (1991). Phase behavior of systems of polyacrylate and cationic surfactants. Langmuir.

[51] Sitar S., Goderis B., Hansson P., Kogej K. (2012). Phase Diagram and Structures in Mixtures of Poly(styrenesulfonate anion) and Alkyltrimethylammonium Cations in Water: Significance of Specific Hydrophobic Interaction. J. Phys. Chem. B.

[52] Kim B., Ishizawa M., Gong J., Osada Y. (1999). Molecular and supramolecular structures of complexes formed by polyelectrolyte-surfactant interactions: Effects of charge density and compositions. J. Polym. Sci. Part A Pol. Chem..

[53] Andersson M., Råsmark P.J., Elvingson C., Hansson P. (2005). Single Microgel Particle Studies Demonstrate the Influence of Hydrophobic Interactions between Charged Micelles and Oppositely Charged Polyions. Langmuir.

[54] Hong W., Liu Z., Suo Z. (2009). Inhomogeneous swelling of a gel in equilibrium with a solvent and mechanical load. Int. J. Solids Struct..

[55] Zhao X., Hong W., Suo Z. (2008). Inhomogeneous and anisotropic equilibrium state of a swollen hydrogel containing a hard core. Appl. Phys. Lett..

[56] Gernandt J., Frenning G., Richtering W., Hansson P. (2011). A model describing the internal structure of core/shell hydrogels. Soft Matter.

[57] Liu Z.-K., Ågren J. (1990). On two-phase coherent equilibrium in binary alloys. Acta Met. Mater..

[58] Doi M. (2009). Gel dynamics. J. Phys. Soc. Jpn..

[59] Edgecombe S., Linse P. (2006). Monte Carlo Simulations of Cross-Linked Polyelectrolyte Gels with Oppositely Charged Macroions. Langmuir.

[60] Jidheden C., Hansson P. (2016). Single Microgels in Core–Shell Equilibrium: A Novel Method for Limited Volume Studies. J. Phys. Chem. B.

[61] Hansson P. (2009). Phase behavior of aqueous polyion–surfactant ion complex salts: A theoretical analysis. J. Colloid Interface Sci..

[62] Horkay F., Tasaki I., Basser P.J. (2001). Effect of monovalent-divalent cation exchange on the swelling of polyacrylate hydrogels in physiological salt solutions. Biomacromolecules.

[63] Hansson P., Bysell H., Månsson R., Malmsten M. (2012). Peptide–Microgel Interactions in the Strong Coupling Regime. J. Phys. Chem. B.

[64] Kokufuta E., Nakaizumi S., Ito S., Tanaka T. (1995). Uptake of sodium dodecylbenzenesulfonate by poly(N-isopropylacrylamide) gel and effect of surfactant uptake on the volume-phase transition. Macromolecules.

[65] Nilsson P., Hansson P. (2005). Ion-Exchange Controls the Kinetics of Deswelling of Polyelectrolyte Microgels in Solutions of Oppositely Charged Surfactant. J. Phys. Chem. B.

[66] Khokhlov A.R., Kramarenko E.Y., Makhaeva E.E., Starodubtzev S.G. (1992). Collapse of polyelectrolyte networks induced by their interaction with an oppositely charged surfactant. Theory. Makromol. Chem. Theory Simul..

[67] Khokhlov A.R., Kramarenko E.Y., Makhaeva E.E., Starodubtsev S.G. (1992). Collapse of polyelectrolyte networks induced by their interaction with oppositely charged surfactants. Macromolecules.

[68] Gernandt J., Hansson P. (2015). Hysteresis in the Surfactant-Induced Volume Transition of Hydrogels. J. Phys. Chem. B.

[69] Al-Tikriti Y., Hansson P. (2020). Drug-Eluting Polyacrylate Microgels: Loading and Release of Amitriptyline. J. Phys. Chem. B.

[70] Schosseler F., Moussaid A., Munch J.P., Candau S.J. (1991). Weakly charged polyelectrolyte gels: Temperature and salt effects on the statics and the dynamics. J. Phys. II Fr..

[71] Zeldovich K.B., Dormidontova E.E., Khokhlov A.R., Vilgis T.A. (1997). Microphase Separation Transition for Polyelectrolyte Gels in Poor Solvents. J. Phys. II Fr..

[72] Gernandt J., Hansson P. (2012). Core–shell separation of a hydrogel in a large solution of proteins. Soft Matter.

[73] Sekimoto K., Kawasaki K. (1989). Elastic instabilities and phase coexistence of gels. Phys. A Stat. Mech. Appl..

[74] Gernandt J., Hansson P. (2016). Surfactant-induced core/shell phase equilibrium in hydrogels. J. Chem. Phys..

[75] Göransson A., Hansson P. (2003). Shrinking Kinetics of Polyacrylate Gels in Surfactant Solution. J. Phys. Chem. B.

[76] Nilsson P., Hansson P. (2007). Deswelling kinetics of polyacrylate gels in solutions of cetyltrimethylammunium bromide. J. Phys. Chem. B.

[77] Bysell H., Malmsten M. (2006). Visualizing the Interaction between Poly-l-lysine and Poly(acrylic acid) Microgels Using Microscopy Techniques: Effect of Electrostatics and Peptide Size. Langmuir.

[78] Andersson M., Hansson P. (2018). Binding of Lysozyme to Spherical Poly(styrenesulfonate) Gels. Gels.

[79] Johansson C., Hansson P., Malmsten M. (2009). Mechanism of Lysozyme Uptake in Poly(acrylic acid) Microgels. J. Phys. Chem. B.

[80] Sasaki S., Koga S. (2002). Elastic Relaxations of Ionic Gel Associated with Hydrophobic Counterion. J. Phys. Chem. B.

[81] Kabanov V.A., Zezin A.B., Rogacheva V.B., Khandurina Y.V., Novoskoltseva O. (1998). Absorption of ionic amphiphils by oppositely charged polyelectrolyte gels. Macromol. Symp..

[82] Karabanova V.B., Rogacheva V.B., Zezin A.B., Kabanov V.A. (1995). Interaction of cross-linked sodium polyacrylate with proteins. Polym. Sci..

[83] Khandurina Y.V., Alexeev V.L., Evmenenko G.A., Dembo A.T., Rogacheva V.B., Zezin A.B. (1995). On the structure of polyacrylate-surfactant complexes. J. Phys. II Fr..

[84] Khandurina Y.V., Dembo A.T., Rogacheva V.B., Zezin A.B., Kabanov V.A. (1994). Structure of polycomplexes composed of cross-linked sodium polyacrylate and cationic micelle-forming surfactants. Polym. Sci..

[85] Skobeleva V.V., Rogacheva V.B., Zezin A.B., Kabanov V.A. (1996). Collapse of swollen gel network and phase transition in a weakly cross-linked polyelectrolyte gel upon its interaction with oppositely charged proteins. Dokl. Phys. Chem..

[86] Zezin A.B., Rogacheva V.B., Kabanov V.A. (1998). Interaction of Linear Polyelectrolytes with Oppositely Charged Lightly Cross-Linked Networks. Macromol. Symp..

[87] Kabanov V.A., Skobeleva V.B., Rogacheva V.B., Zezin A.B. (2004). Sorption of Proteins by Slightly Cross-Linked Polyelectrolyte Hydrogels: Kinetics and Mechanism. J. Phys. Chem. B.

[88] Chu B., Yeh F., Sokolov E.L., Starodoubtsev S.G., Khokhlov A.R. (1995). Interaction of Slightly Cross-Linked Gels of Poly(diallyldimethylammonium chloride) with Surfactants. Macromolecules.

[89] Dembo A.T., Yakunin A.N., Zaitsev V.S., Mironov A.V., Starodoubtsev S.G., Khokhlov A.R., Chu B. (1996). Regular microstructures in gel-surfactant complexes: Influence of water content and comparison with the surfactant structure in water. J. Polym. Sci. Part B Polym. Phys..

[90] Sokolov E.L., Yeh F., Khokhlov A., Chu B. (1996). Nanoscale Supramolecular Ordering in Gel−Surfactant Complexes: Sodium Alkyl Sulfates in Poly(diallyldimethylammonium Chloride). Langmuir.

[91] Yeh F., Sokolov E.L., Khokhlov A.R., Chu B. (1996). Nanoscale Supramolecular Structures in the Gels of Poly(Diallyldimethylammonium Chloride) Interacting with Sodium Dodecyl Sulfate. J. Am. Chem. Soc..

[92] Yeh F., Sokolov E.L., Walter T., Chu B. (1998). Structure Studies of Poly(diallyldimethylammonium chloride-co-acrylamide) Gels/Sodium Dodecyl Sulfate Complex. Langmuir.

[93] Zhou S., Burger C., Yeh F., Chu B. (1998). Charge Density Effect of Polyelectrolyte Chains on the Nanostructures of Polyelectrolyte−Surfactant Complexes. Macromolecules.

[94] Zhou S., Yeh F., Burger C., Chu B. (1999). Nanostructures of polyelectrolyte gel-surfactant complexes. J. Polym. Sci. Part B Polym. Phys..

[95] Ashbaugh H.S., Lindman B. (2001). Swelling and Structural Changes of Oppositely Charged Polyelectrolyte Gel−Mixed Surfactant Complexes. Macromolecules.

[96] Hansson P. (1998). Surfactant Self-Assembly in Polyelectrolyte Gels: Aggregation Numbers and Their Relation to the Gel Collapse and the Appearance of Ordered Structures in the NaPA/C12TAB System. Langmuir.

[97] Nilsson P., Unga J., Hansson P. (2007). Effect of Salt and Surfactant Concentration on the Structure of Polyacrylate Gel/Surfactant Complexes. J. Phys. Chem. B.

[98] Sasaki S., Koga S., Sugiyama M., Annaka M. (2003). Nanostructures of polyelectrolyte gel-surfactant complexes in uniaxially stretched networks. Phys. Rev. E.

[99] Svensson A., Topgaard D., Piculell L., Söderman O. (2003). Molecular Self-Diffusion in Micellar and Discrete Cubic Phases of an Ionic Surfactant with Mixed Monovalent/Polymeric Counterions. J. Phys. Chem. B.

[100] Svensson A., Norrman J., Piculell L. (2006). Phase behavior of polyion-surfactant ion complex salts: Effects of surfactant chain length and polyion length. J. Phys. Chem. B.

[101] Norrman J., Lynch I., Piculell L. (2007). Phase Behavior of Aqueous Polyion-Surfactant Ion Complex Salts: Effects of Polyion Charge Density. J. Phys. Chem. B.

[102] Norrman J., Piculell L. (2007). Phase Behavior of Cetyltrimethylammonium Surfactants with Oligo Carboxylate Counterions Mixed with Water and Decanol: Attraction between Charged Planes or Spheres with Oligomeric Counterions. J. Phys. Chem. B.

[103] Janiak J., Schillén K., Piculell L., Olofsson G. (2011). The aqueous phase behavior of polyion–surfactant ion complex salts mixed with nonionic surfactants. Phys. Chem. Chem. Phys..

[104] Thalberg K., Lindman B., Karlstroem G. (1990). Phase diagram of a system of cationic surfactant and anionic polyelectrolyte: Tetradecyltrimethylammonium bromide-hyaluronan-water. J. Phys. Chem..

[105] Liang J., Xiao X., Chou T.-M., Libera M. (2019). Counterion Exchange in Peptide-Complexed Core–Shell Microgels. Langmuir.

[106] Råsmark P.J., Andersson M., Lindgren J., Elvingson C. (2005). Differences in Binding of a Cationic Surfactant to Cross-Linked Sodium Poly(Acrylate) and Sodium Poly(Styrene Sulfonate) Studied by Raman Spectroscopy. Langmuir.

[107] Hansson P. (2009). Surfactant Self-Assembly in Oppositely Charged Polymer Networks. Theory. J. Phys. Chem. B.

[108] Nilsson P., Hansson P. (2008). Regular and irregular deswelling of polyacrylate and hyaluronate gels induced by oppositely charged surfactants. J. Colloid Interface Sci..

[109] Ahnfelt E., Gernandt J., Al-Tikriti Y., Sjögren E., Lennernäs H., Hansson P. (2018). Single bead investigation of a clinical drug delivery system—A novel release mechanism. J. Control. Release.

[110] Ahnfelt E., Sjögren E., Hansson P., Lennernäs H. (2016). In Vitro Release Mechanisms of Doxorubicin From a Clinical Bead Drug-Delivery System. J. Pharm. Sci..

[111] Bysell H., Hansson P., Malmsten M. (2008). Transport of poly-l-lysine into oppositely charged poly(acrylic acid) microgels and its effect on gel deswelling. J. Colloid Interface Sci..

[112] Bysell H., Hansson P., Malmsten M. (2010). Effect of Charge Density on the Interaction between Cationic Peptides and Oppositely Charged Microgels. J. Phys. Chem. B.

[113] Bysell H., Hansson P., Schmidtchen A., Malmsten M. (2010). Effect of Hydrophobicity on the Interaction between Antimicrobial Peptides and Poly(acrylic acid) Microgels. J. Phys. Chem. B.

[114] Bysell H., Schmidtchen A., Malmsten M. (2009). Binding and Release of Consensus Peptides by Poly(acrylic acid) Microgels. Biomacromolecules.

[115] Carta G., Ubiera A.R., Pabst T.M. (2005). Protein Mass Transfer Kinetics in Ion Exchange Media: Measurements and Interpretations. Chem. Eng. Technol..

[116] Biondi M., Fusco S., Lewis A.L., Netti P.A. (2012). New Insights into the Mechanisms of the Interactions Between Doxorubicin and the Ion-Exchange Hydrogel DC Bead™ for Use in Transarterial Chemoembolization (TACE). J. Biomater. Sci. Polym. Ed..

